# Colonization by *Akkermansia muciniphila* modulates central nervous system autoimmunity in an ecological context-dependent manner

**DOI:** 10.3389/fimmu.2025.1655428

**Published:** 2025-10-13

**Authors:** Daniel Peipert, Theresa L. Montgomery, Lucinda C. Toppen, Margaret Frances J. Lee, Matthew J. Scarborough, Dimitry N. Krementsov

**Affiliations:** ^1^ Department of Biomedical and Health Sciences, University of Vermont, Burlington, VT, United States; ^2^ Department of Civil and Environmental Engineering, University of Vermont, Burlington, VT, United States; ^3^ Department of Microbiology and Molecular Genetics, University of Vermont, Burlington, VT, United States

**Keywords:** *Akkermansia muciniphila*, experimental autoimmune encephalomyelitis (EAE), gut microbiome, multiple sclerosis (MS), short-chain fatty acid (SCFA), fiber, metabolites

## Abstract

**Introduction:**

Multiple sclerosis is autoimmune disease of the central nervous system (CNS) in which myelin-reactive immune attack drives demyelination and subsequent disability. Various studies have documented elevated abundance of the commensal gut bacterium *Akkermansia muciniphila* (*A. muciniphila*) in people with multiple sclerosis compared to healthy control subjects, suggesting that its elevated abundance may be a risk factor for the development of CNS autoimmunity. However, *A. muciniphila* is considered beneficial in various other pathological contexts, and recent studies suggest that *A. muciniphila* may be paradoxically associated with reduced disability and progression in multiple sclerosis. Moreover, experimental modulation of *A. muciniphila* levels in experimental autoimmune encephalomyelitis (EAE), an autoimmune model of multiple sclerosis, has generated conflicting results, suggesting that the effects of this microbe on CNS autoimmunity could be context-dependent.

**Methods:**

To address this possibility, we generated two distinct microbiome models in C57BL/6J mice, each stably colonized by *A. muciniphila* or *A. muciniphila*-free, providing divergent ecological contexts in which *A. muciniphila* may exert a differential impact. We used EAE, flow cytometry, full-length 16S DNA sequencing, and mass spectrometry to assess the impact of *A. muciniphila* colonization on neurological outcomes, immune responses, gut microbiome composition, and short-chain fatty acid (SCFA) production, respectively. Dietary intervention was used to assess the functional consequences of differences in gut microbiota metabolic capacity.

**Results:**

We found that *A. muciniphila* colonization increased EAE severity only in a specific microbiome context, in conjunction with increased Th17 responses and CNS-infiltrating immune cells. Profiling of gut microbiome composition revealed that *A. muciniphila* colonization drove a reduction of *Clostridia*, key producers of SCFAs, specifically in the microbiome model in which *A. muciniphila* exacerbates EAE. Inferred metagenomic analyses suggested reduced SCFA production in the presence of *A. muciniphila*, which was confirmed by mass spectrometry. Consistently, provision of high dietary fiber as a substrate for SCFA production suppressed EAE only in the context of the *Clostridia*-rich microbiome sensitive to *A. muciniphila* colonization.

**Discussion:**

Taken together, our data suggest that the effect of *A. muciniphila* on CNS autoimmunity is highly dependent on the overall composition of the gut microbiome and suggest that this microbe may contribute to decreased gut SCFA metabolism in multiple sclerosis.

## Graphical Abstract

Leveraging divergent gut microbiome backgrounds, we assessed the severity of experimental autoimmune encephalomyelitis and the microbiome composition caused by experimental colonization by *A. muciniphila*. *A. muciniphila* colonization in the Clostridia-rich microbiome (depicted on the right side), but not in a Clostridia-low microbiome (depicted on the left) contributed to worsened disease severity, an exacerbated Th17 immune response, depletion of Clostridia, and a reduction in SCFAs.

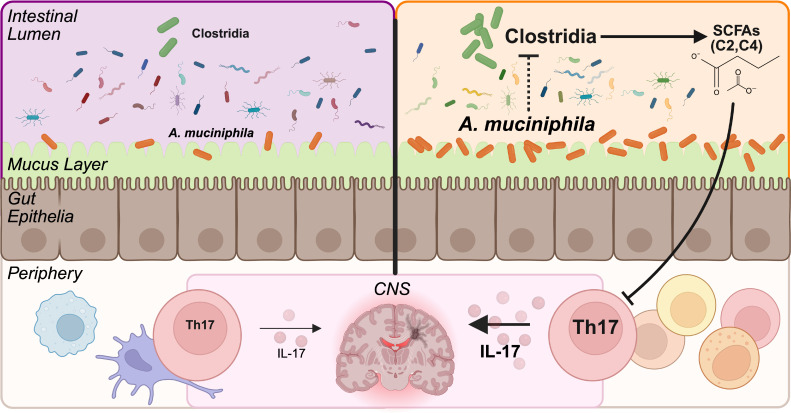

## Introduction

Multiple sclerosis (MS) is the most common demyelinating and nontraumatic neurological disorder among young adults, affecting ~2.8 million individuals globally (1, 2). In MS, immune cells infiltrate into the central nervous system (CNS) and mount an autoimmune response against myelin, leading to demyelination, axonal loss, and neurological dysfunction (3). People with MS (pwMS) can experience a myriad of cognitive, sensory, and motor symptoms (4). At the cellular level, peripheral myelin-specific CD4^+^ T cells migrate across the blood brain barrier into the CNS and drive MS disease, with Th1 and Th17 cells being implicated in disease initiation and progression (3). CNS myelin autoreactivity and subsequent neuropathology can be modeled using experimental autoimmune encephalomyelitis (EAE), by immunizing mice with myelin peptides to elicit CNS demyelination and neurological disability akin to some symptoms experienced by pwMS (5).

Research on the etiology of MS has emphasized that while genetics impart a consequential portion of disease susceptibility, environmental factors, including the gut microbiome, contribute significantly to disease risk and progression (6–10). Perturbations in the gut microbiome are of growing interest in understanding underlying MS risk factors, pathology, treatment, and prognosis. A key function of the gut microbiome is the production of various bacteria-derived secondary metabolites, including short-chain fatty acids (SCFAs), that can have immunological consequences for the host (9, 10). Multiple studies have identified changes in the gut microbiome correlated with MS disease status and progression (11). These studies have highlighted two major consistent phenotypes associated with pwMS compared to healthy controls: an increased abundance of *Akkermansia muciniphila* and a reduction in SCFA-producing bacteria (12–17). Increased abundance of *A. muciniphila* is associated with MS, as documented by numerous studies (18–20), making it a key species of relevance to the MS microbiome. Paradoxically, higher levels of *A. muciniphila* in pwMS are associated with lower disease severity or progression (18, 21). However, beyond these associations, causal and mechanistic relationships between specific gut bacteria and MS pathophysiology are lacking.

*A. muciniphila* is a commensal gut bacterium capable of metabolizing host mucin, and it can represent up to 3% of the total gut bacteria in humans (22, 23). It has been negatively associated with a variety of metabolic human conditions, including type 2 diabetes, obesity, and cardiovascular disease (24–26). In contrast, it is elevated in MS and Parkinson’s disease and altered in other neurological conditions (27–29). While *A. muciniphila* has been shown to produce the SCFAs, acetate (C2) and propionate (C3), from host mucin *in vitro (* 22, 30, 31), it remains unclear whether this mucin degradation or acetate and propionate production are consistent across microbiome contexts and/or sufficient to affect CNS autoimmunity in the host. Other studies have failed to find elevated SCFAs associated with *A. muciniphila* in gut microbiome models *in vivo*, suggesting instead that its effects on SCFA levels may be microbiome specific, diet specific, and/or indirect (i.e. via other microbes) (32–34).

Similar to the human studies described above, animal models have suggested opposing roles for *A. muciniphila*. Akin to pwMS, the abundance of *A. muciniphila* is elevated in mice with EAE, and daily gavage with high doses of *A. muciniphila* as a therapeutic intervention suppresses EAE severity (18, 19, 35). However, this experimental approach, while informative for a probiotic strategy, does not appropriately model complex interactions among endogenous and stably colonized commensal bacteria that are typically assessed in observational studies of disease in humans (36–38). In contrast to therapeutic intervention, stable colonization by *A. muciniphila* has been associated with increased EAE severity (39, 40), emphasizing the difference between treatment and colonization experimental approaches, and/or the importance of ecological context (i.e. well-known differences in gut microbiota composition across different mouse colonies) (41, 42). Altogether, the role of *A. muciniphila* in the context of MS warrants further exploration to better understand its role in disease pathophysiology, as a potential biomarker, and/or for guiding preventative measures and therapeutics.

Within the mammalian host, SCFAs are thought to exert beneficial effects by modulating neuronal activity, supporting blood brain barrier integrity, and producing anti-inflammatory IL-10 and T regulatory responses (43–47). Butyrate is also relevant to gut mucus homeostasis and has been shown to increase mucin gene expression in goblet and epithelial cells (48). SCFA concentrations are depleted in the sera and feces of pwMS compared to healthy controls, and SCFAs have been broadly associated with reduced progression of disability (13, 20, 49, 50). Bacteria within the class *Clostridia*, including *Lachnospiraceae*, *Ruminococcaceae*, and *Oscillospiraceae*, are known to produce butyrate and other SCFAs from dietary fiber (51–55). Case control studies on pwMS and healthy controls also demonstrate a reduction in SCFA-producing *Clostridia* species associated with MS (13, 20, 56), and a reduction in *Clostridia* is similarly observed after EAE induction (57). Like studies on *A. muciniphila* gavage treatment, treatment with serial gavages of *Clostridia* in EAE mice reduced disease severity and increased serum butyrate concentrations (58, 59). Supplementation of individual SCFAs, SCFA cocktails, and prebiotic dietary fiber have been shown to ameliorate disease severity in EAE models (60–62), although evidence in support of SCFA supplementation for pwMS is so far preliminary (63). Importantly, factors that drive the reduction in SCFA-producing bacteria in pwMS are not understood.

Microbe-microbe interactions are critical to understanding how the gut microbiome influences host physiology. These interactions can include collaboration, where bacterial cross-feeding contributes to secreted metabolites (64–66), and competition, where species may sequester nutrients and/or produce small molecule antagonists that target other bacteria (67–69). Specifically, *A. muciniphila* contributes to trophic interactions by liberating host mucin glycans that may be catabolized by other gut bacteria (40, 70–74). These interactions emphasize the importance of the broader gut microbiome ecological context as a key unaddressed variable in human and animal studies on CNS autoimmunity.

Previous work in our lab has established compositionally complex yet reproducible gut microbiome models by colonizing germ-free C57BL/6J (B6) hosts with cecal microbiota from specific pathogen-free (SPF) B6 and wild-derived and genetically divergent Prague wild derived D (PWD) mice, generating genetically identical hosts with divergent microbiomes (75). Here, we utilized these models to isolate the effects of ecological context on *A. muciniphila* colonization and its subsequent consequences for the host. Given that *A. muciniphila* is an endogenous commensal in humans rather than an exogenous therapeutic, we focused on colonization approaches to better understand how *A. muciniphila* can predispose to or protect against subsequent disease development and/or severity. Our data demonstrate that *A. muciniphila* colonization increases EAE severity in a highly microbiome context-specific manner. 16S analyses demonstrated that EAE exacerbation by *A. muciniphila* is coupled with a reduction in the abundance of *Clostridia* and SCFA production potential, all of which are microbiome-context dependent. Functionally, a high fiber diet selectively ameliorated EAE severity in mice harboring *Clostridia*-rich microbiome, emphasizing the dependency of this microbiome on gut SCFAs and its susceptibility to *A. muciniphila*-mediated EAE exacerbation by a reduction in *Clostridia*. Together, our results demonstrate that the effects of *A. muciniphila* on CNS autoimmunity are highly dependent on interactions with other members of the gut microbiota, suggesting that *A. muciniphila’s* use as a biomarker or therapeutic in pwMS will require assessment of the full microbiome composition as a key covariate.

## Materials and methods

### Animals

All experimental procedures used in this study were approved by the University of Vermont’s Animal Care and use Committee. Mice were maintained under barrier conditions with sterilized caging, fed irradiated diets (Prolab IsoPro RMH 3000), and handled minimally in a structured sequence to avoid cross-contamination and the introduction of new microbes. Donor male C57BL/6J (B6) and PWD/PhJ (PWD) mice used for cecal microbiota transplant were purchased from Jackson Laboratories (Bar Harbor, Maine, USA) and housed within a single vivarium room at the Larner College of Medicine at the University of Vermont for 2–4 generations. Gut microbial transplantation from cecal donors was performed as previously described (75); ceca from donor B6 and PWD mice were collected and transferred to an anaerobic chamber where the contents were flushed out and mixed to a final concentration of 20% glycerol in Hungate tubes, flash frozen, and stored at -80 °C in single use aliquots. PWDβ cecal stocks were generated by collecting and combining cecal contents of ex-germ free B6 mouse recipients of the PWD microbiome, and pups of ex-germ free B6 mouse recipients of the PWD microbiome, performed as above.

Germ-free (GF) 7–9 week-old B6 mice from the National Gnotobiotic Rodent Resource Center at the University of North Carolinae School of Medicine (USA) were shipped in sterile crates. GF B6 mice were opened under a laminar flow hood and immediately inoculated by gastric gavage with 100-200 µl of cryopreserved stocks from B6 or PWD ceca, generating B6.Gut^B6^ mice (denoted as “B6”) and B6.Gut^PWD^ mice (denoted as “PWD”), respectively. Colonization by *A. muciniphila* was achieved in B6 and PWD microbiome-colonized mice with a series of 3 gastric gavages of 1.65×10^7^ CFU/200 µl *A. muciniphila* every other day, generating B6.Gut^B6+Akk^ mice (denoted as “B6+Akk”) and B6.Gut^PWD+Akk^ mice (denoted as “PWD+Akk”). B6.Gut^PWDβ^ mice (“PWDβ”) were generated using a single 200 µl gastric gavage containing 100 µl of cryopreserved stocks from PWDβ cecal stock and 100 µl of anerobic 50% glycerol in PBS. B6.Gut^PWDβ+Akk^ counterparts (denoted as “PWDβ+Akk”) were generated using a single 200 µl gastric gavage containing 100 µl of cryopreserved PWDβ cecal stock and 8.26x10^6^ CFU/100 µl of *A. muciniphila*. The resulting B6, B6+Akk, PWD, PWD+Akk, PWDβ, and PWDβ+Akk mice were paired for breeding to generate male and female pups with vertically transmitted gut microbiota to be used for subsequent experiments.

For dietary fiber intervention experiments, mice were randomized by microbiome to receive low fiber (TD.180916, 0% fermentable fiber) or high fiber (TD.220544, 20% inulin, 10% pectin) for 2 weeks prior to EAE induction. Low and high fiber chow (Inotiv, USA) were vacuum packed, irradiated, and stored at 4°C. Chow was refreshed daily.

### EAE

EAE was induced in 6-11-week-old pups of specific microbiota-colonized ex-germ-free (ex-GF) breeders using the 2× MOG_35-55_/CFA protocol as previously described (76); mice were injected subcutaneously with 100 µl of emulsion containing 100 µg myelin oligodendrocyte glycoprotein 35-55 (MOG_35-55_; New England Peptide, USA) in 50% complete Freund adjuvant (CFA; Sigma, USA) supplemented with an additional 4 mg/ml *Mycobacterium tuberculosis* H37Ra (Difco, USA). A first set of injections was completed on each lower flank (50 µl/side), and a 2^nd^ set of injections was completed 7 days later into each upper flank (50 µl/side) of the mouse. On days 10 through 30, mice were scored for ascending paralysis as follows: 0 – asymptomatic, 1 – loss of tail tone, 2 – loss of tail tone and hind limb weakness, 3 – hind limb paralysis, 4 – hind limb paralysis with incontinence, and 5 – moribund/quadriplegic. Cumulative disease score was calculated as the sum of all daily scores. Cages that included at least one mouse exhibiting hind limb paralysis received 2–3 chow pellets and approximately 2cm^3^ of Napa nectar (Systems Engineering, USA) on the cage floor, both refreshed daily.

### Microbial DNA isolation and species-specific qPCR

Fecal samples were collected by transferring individual mice to empty cages without bedding and waiting for them to defecate at least one fecal pellet. Collection cages were only used within-microbiome group and replaced for each experiment. Once collected, pellets were kept on ice before being stored at -80 °C until later use. DNA was extracted from fecal pellets using QIAamp PowerFecal Pro DNA extraction kits (Qiagen, USA); DNA quality and quantity was assessed via Nanodrop. *A. muciniphila* relative abundance was quantified using species-specific primers (forward sequence, CAGCACGTGAAGGTGGGGAC; reverse sequence, CCTTGCGGTTGGCTTCAGAT) normalized to a pan-bacteria eubacteria primer set (forward sequence, ACTCCTACGGGAGGCAGCAG; reverse sequence, ATTACCGCGGCTGCTGG). Quantification by qPCR was achieved with Dynamo ColorFlash SYBR Green (Thermo Fisher Scientific, USA) on a Quant Studio 3 or 5 Real-Time PCR machine (Thermo Fisher Scientific, USA) with annealing temperatures of 66 °C for *A. muciniphila* and 60 °C for eubacterial primers and 30 PCR cycles. Any B6+Akk, PWD+Akk, and PWDβ+Akk mice whose fecal samples did not show amplification for *A. muciniphila* prior to EAE induction were assumed to be unsuccessfully colonized and thus excluded from the analysis. *A. muciniphila*-free B6, PWD, and PWDβ feces were also tested for *A. muciniphila*, which was never observed in these samples. Melting curves were also used to confirm the presence of a single peak at 87 °C for detecting *A. muciniphila*.

### Lipocalin-2 ELISA

Fecal slurries were prepared from frozen fecal samples. Fecal pellets were weighed and transferred to a screw-top 2 mL tube filled approximately 1/5 full of 1.0 mm diameter silicon carbide sharp particles (BioSpec, USA). Cold phosphate-buffered saline (PBS) with 0.01% Tween 20 was added to achieve 50 mg feces/mL. Tubes were homogenized by vortexing with a tube adapter for 10 minutes and pelleted at 15,000 × g for 10 minutes. The supernatant was transferred to a clean tube and stored for use. Lipocalin-2 was quantified using ELISA reagent kits per manufacturers procedure (R&D Systems, USA). Optical density was measured at 450 nm with background subtraction at 570 nm wavelength and concentrations were calculated using a standard curve. PBS with 0.01% Tween 20 was used for washing, and PBS with 1% bovine serum albumin (Sigma, USA) was used for blocking and reagent dilutions.

### 16S DNA sequencing and preprocessing

Fecal DNA, extracted as described above, was utilized for full length 16S ribosomal RNA gene amplicon sequencing at the University of Illinois W.M. Keck Center for Comparative and Functional Genomics. 16S amplicons were generated with barcoded primers from PacBio targeting the entire 16S gene (forward sequence, AGRGTTYGATYMTGGCTCAG; reverse sequence, RGYTACCTTGTTACGACTT) and Roche KAPA HiFi Hot Start Ready Mix. The amplicons were converted into a sequencing library with a SMRTbell Express Template Prep Kit 3.0, and the library was then sequenced on a single SMRTcell 8M using a PacBio Sequel IIe platform in Circular Consensus Sequencing (CCS) mode and a 15-hour movie run time. CCS libraries were analyzed for read quality and demultiplexed on SMRTLink version 11.1, creating raw sequencing reads as FASTQ files for each fecal sample.

The raw FASTQ sequencing files were analyzed in R Studio (R version 4.2.1) with preprocessing using the *DADA2* package (version 1.26.0) (77). Following primer removal, reads were filtered to include only those with a nucleotide length between 1000 and 1600, a minimum quality score of 3, a maximum number of expected errors (maxEE) of 2. Dereplicated data was used to generate and apply an error model, followed by denoising and then removal of chimeric sequences by the consensus method, generating amplicon sequencing variants (ASVs). ASV sequences were reference against SILVA database (version 138.1) for taxonomic assignment (78, 79). Construction of a phylogenetic tree was done using the *DECIPHER* package (version 2.26.0) for alignment of unaligned sequences (80) and the *phangorn* package (version 2.11.1) for building the tree using the neighbor-joining method with pairwise computed distances, a general time-reservable model, the nearest neighbor interchange, and rooted using a randomly selected ASV (81). The resulting phylogenetic tree, ASV table, ASV sequences, taxonomic assignments, and imported metadata were combined in *phyloseq* (version 1.48.0) to create a *phyloseq* object for downstream analyses (82).

### Analysis of 16S data

Alpha diversity, determined by Shannon index, was graphed and calculated using the *phyloseq* functions plot_richness and estimate_richness and compared by Wilcox test with Holm-Bonferroni correction for multiple comparisons. For subsequent analyses, ASVs within the phyloseq object were first agglomerated using the tip_glom function to a height threshold of 0.05; using the metagMisc package (version 0.0.4), ASVs were then filtered to only include those with a prevalence of at least 3% and a mean abundance threshold of 2. Graphical representations of 16S data, including the relative abundance of bacterial taxa across samples, were generated using the constructed *phyloseq* object and *ggplot2* (version 3.5.1). Beta diversity was calculated using the ordinate and plot_ordination functions of *phyloseq*, represented using PCoA plots, and compared across microbiome groups by permutation multivariate ANOVA using the adonis function from the *vegan* package (version 2.6-4) (83) and the pairwise adonis function from the *pairwiseAdonis* package (version 0.4.1). Differential abundance was calculated with the *DESeq2* package (version 1.38.3) using Wald significance testing and an adjusted p <0.05 (70). Composition networks of microbiome structures were created using the *NetCoMi* package (version 1.1.0) using the Semi-Parametric Rank-based approach for INference in Graphical (SPRING) model that included only the 50 most abundant ASVs and with a sparsity parameter (lambda) of 20 and filtering to the 50 most frequent reads (84).

### Functional pathway analysis

Inferred functional metagenomic pathways of the entire gut microbiota of each sample was mapped using PICRUSt2 (version 2.2.0_b) in Python (version 3.6.7) (85). The abundance of inferred pathways was then applied to a new *phyloseq* object as a wrapper and analyzed using *DESeq2* as done with ASVs described above. ASV contributions to individual enzyme commission (EC) numbers were generated in PICRUSt2 to analyze individual ASV contributions to butyrate production as previously described (86). Butyrate producers were defined as bacteria conserving any of the following terminal enzymes in butyrate-producing pathways: butyryl-CoA:4-hydroxybutyrate CoA transferase (4Hbt; EC:2.8.3.-), butyryl-CoA:acetoacetate CoA transferase (Ato; EC:2.8.3.9), butyryl-CoA: acetate CoA transferase (But; EC:2.8.3.8) or butyrate kinase (Buk; EC:2.7.2.7) (87). Statistical differences in relative abundance of all butyrate producer ASVs between comparisons was determined by Wilcoxon rank sum non-parametric testing.

### Bacterial culture

**Figure 1:**
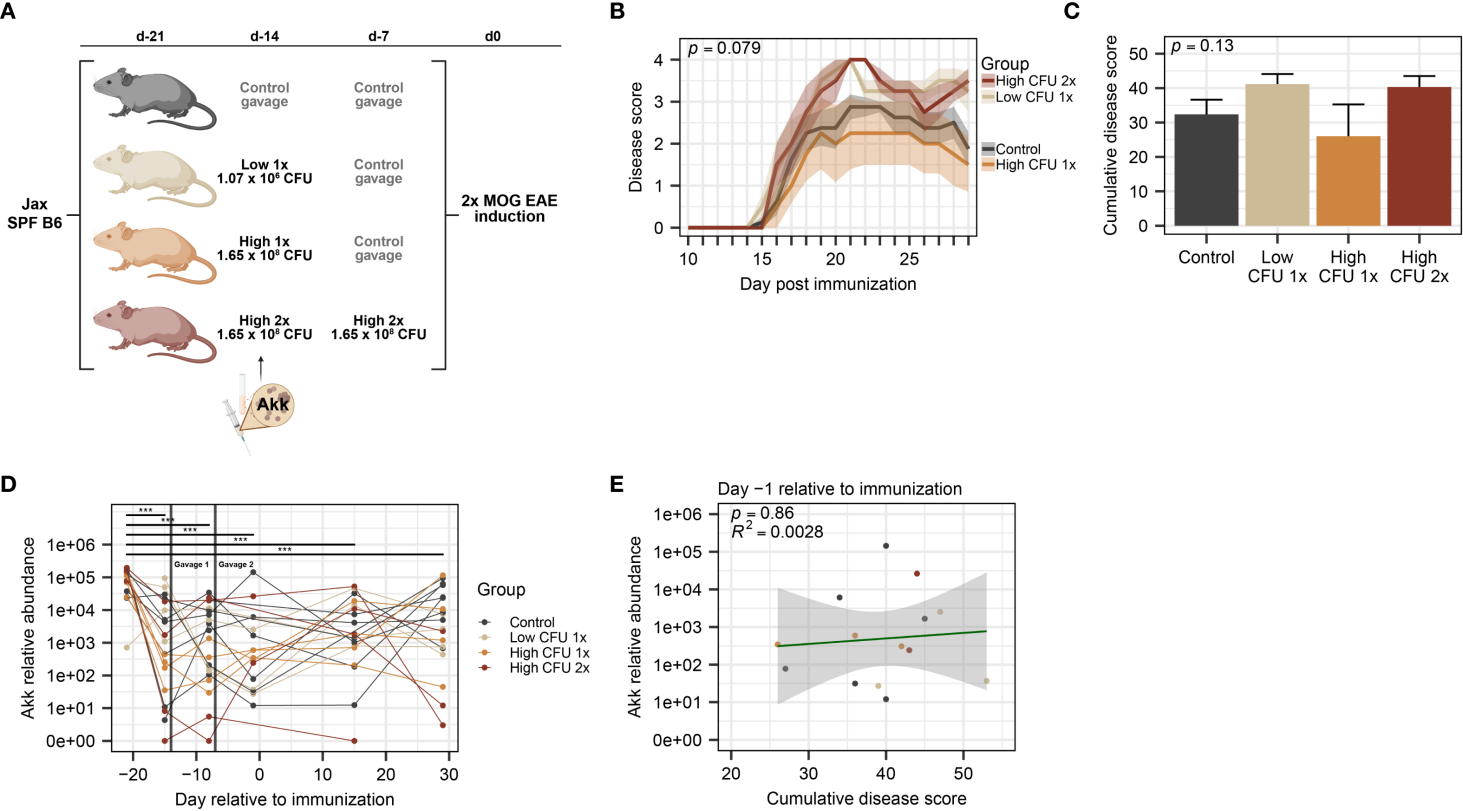
Treatment with *A*. *muciniphila* by oral gavage in commercially available mice does not modulate EAE severity. SPF B6 mice obtained from Jax were divided among treatment groups: a control group (n=8, all male) that received vehicle and 3 groups (n=4 per group) that received various amounts of cryopreserved *A*. *muciniphila* culture 14 and 7 days prior to EAE induction, as indicated. **(A)** Schematic of experimental design indicating treatment dosage and timing. Mice were received at D-21 and rested until treatment by oral gavage at D-14 and D-7, as indicated, followed by EAE induction at D0. **(B)** Clinical course of EAE, as analyzed by two-way ANOVA, with significance representing the time × treatment interaction. **(C)** EAE cumulative disease scores, analyzed by one-way ANOVA. **(D)** Relative abundance of *A*. *muciniphila* DNA across timepoints (n=2–8 per group-timepoint), measured by species-specific qPCR, was analyzed using a linear mixed-effects model and analyzed via ANOVA with Holm-Bonferroni-corrected multiple comparisons across all groups (*** indicates *p* < 0.001). **(E)** *A*. *muciniphila* abundance at D-1 versus cumulative disease score (n=2–6 per group), assessed by linear regression with p-value indicating significance of association and R^2^ indicating goodness of fit.

*A. muciniphila* murine strain YL44/DSM26127 was provided by Dr. Adam Sateriale (currently at the Francis Crick Institute, UK) and was originally obtained from DSMZ (Leibniz Institute, Germany). *A. muciniphila* culture used for gastric gavages was grown anaerobically in brain heart infusion broth (BHI; Sigma, USA) supplemented with 15% of fetal bovine serum (Thermo Fisher Scientific, USA), 5 g/L yeast extract (Sigma, USA), 0.2 mL/L vitamin K solution (Sigma, USA; made as 1% vitamin K1 in 100% ethanol), 0.5 mL/L of hemin solution (Sigma, USA; 0.5g/L dissolved in 1% NaOH solution), 0.5 g/L cysteine (Sigma, USA). Cultures were grown in 5 mL volumes of liquid BHI with 100 µL of 5% type 3 porcine stomach mucin (Sigma, USA; 50 g/L mucin mixed in deionized water). *A. muciniphila* was grown without shaking to an OD_600_ of 0.833, used as high-density stocks and for breeding pair mice (B6+Akk, PWD+Akk, and PWDβ+Akk) and an OD_600_ of 0.215, then diluted 1:10 using bacteria-free media, for low-density stocks. CFU was calculated using an OD_600_ 0.8 equivalent of 1x10^8^ CFU/mL of *A. muciniphila* after subtracting the OD_600_ of the growth media (88). Anaerobic conditions were maintained in an anerobic chamber (Coy Labs, USA) with 5% carbon dioxide, 2% hydrogen, and 91% nitrogen at 37 °C. Individual cultures were pooled prior to being cryopreserved for future use as gavage stocks, which were made as 50% liquid culture, 25% sterile glycerol (Sigma, USA), and 25% sterile PBS. For treatment of control group SPF B6 mice (**Figure 1A**), a vehicle containing 50% media, 25% sterile glycerol, and 25% sterile PBS was used.

### Flow cytometry

Post-EAE mice were anaesthetized with isoflurane (Piramal, USA) and perfused transcardially with 40 mL of cold PBS. Each spinal cord was dissected, homogenized using a Dounce glass homogenizer, and filtered through a 70 µm strainer. The resulting single-cell suspension was mixed with 37% Percoll (Cytiva, USA) and loaded above 70% Percoll to achieve a Percoll gradient that isolated mononuclear cells from the interphase following centrifugation. Isolated cells were stimulated with 5 ng/mL PMA, 250 ng/mL ionomycin, and brefeldin A (GolgiPlug; BD Bioscience, USA) for 4 hours. Cells were then labeled with UV-Blue Live/Dead (Thermo Fisher Scientific, USA), stained with surface antibodies: CD45, CD11b, CD19, CD4, CD8, TCRβ, TCRγδ (BioLegend, USA), fixed and permeabilized with 0.2% saponin (Sigma, USA), then labeled with intracellular antibodies: IL-17A and IFNγ (BioLegend, USA). Stained cells were analyzed in the Harry Hood Bassett Flow Cytometry and Small Particles Detection facility (RRID: SCR_022147) at the Larner College of Medicine using a Cytek Aurora and SpectroFlo software (versions 2.2-3.3; Cytek Biosciences, USA) using spectral unmixing with single-color controls and autofluorescence correction from unstained cells. Data was analyzed using FlowJo software (version 10.10.0) (BD Biosciences).

### SCFA quantification

Gas chromatography coupled with mass spectrometry (GC-MS) was used to quantify SCFA levels in fecal samples, including acetate, propionate, butyrate, and isobutyrate. Fecal samples were directly weighed into a 2 mL microcentrifuge tube, and 700 mg silica beads and 600 µL distilled and deionized water were added to each. Samples were homogenized using a bead beater. After homogenization, samples were centrifuged at 10,000 rpm for 10 minutes and the liquified sample in the supernatant was extracted. Solvent extraction of SCFAs was performed by combining 200 µL of supernatant, 20 µL of hydrochloric acid (HCl), 100 µL of potassium bisulfate (KHSO4), and 1 mL of dimethyl carbonate (DMC) into a 2 mL microcentrifuge tube (89). Upon addition of HCl, the anion forms of SCFAs are protonated such that they readily partition into the DMC. The resulting mixture was vortexed for 10 seconds and centrifuged at 3800 rpm for 10 minutes. The supernatant organic phase was then transferred into a GC-MS vial for analysis. For the quantification, a Shimadzu Nexus GS 2030 couple to a TQ8040NX mass spectrometer was used. The GC-MS autosampler used a 2 μL smart syringe to inject 0.8 μL of liquid sample. A DB-FATWAX UI column, with 30 m length, 0.25 μm thickness, and 0.25 mm diameter was used. Upon injection of the sample, the oven temperature was held at 80 °C for 1 minute and then increased by 15 °C/minute until it reached 115 °C. The oven temperature was held at 115 °C for 3 minutes. Then, the oven temperature ramped again at a rate of 3 °C/minute until it reached 130 °C. The temperature was then increased at a rate of 15 °C/minute until it reached 230 °C. The oven was held at 230 °C for 3 min. Acquisition mode was Q3 scan, with ion source temperature and interface temperature at 280 °C and 250 °C, respectively. Concentrations were determined using a calibration curve generated from external standards that underwent the same extraction protocol as samples. Concentrations were then used to calculate mg of each SCFA per gram of fecal matter.

## Results

### Oral gavage with *A. muciniphila* fails to increase its abundance and modulate EAE severity in commercially available B6 SPF mice, which are colonized by endogenous *A. muciniphila*

To assess a potential causative role for *A. muciniphila* in modulating EAE severity, we sought to modulate its abundance by colonization via oral gavage, first using commercially available SPF C57BL/6J (B6) mice from the Jackson Laboratory (Jax). B6 Jax mice were assigned to gavage treatment groups and inoculated by a single oral gavage with 200µl of *A. muciniphila* culture from either low (1.07×10^6^ CFU) or high-density (1.65×10^8^ CFU) anaerobically grown stocks, and a 3^rd^ treatment group received two gavages of high-density culture (**Figure 1A**), based on previous studies suggesting that 1× and 2× oral gavage of *A. muciniphila* is sufficient to modulate its abundance in SPF mice (90, 91). Sterile vehicle control was administered as a 2^nd^ gavage for single-gavage-treated groups and control groups. EAE was induced at 7 or 14 days post-treatment (see **Figure 1A**) by immunization with MOG_35–55_ in CFA, as previously described.

Analysis of *A. muciniphila* treatment effect on EAE disease course revealed no significant differences across gavage groups or any dose-dependent trends (**Figure 1B**). Similarly, analysis of cumulative disease score demonstrated no significant differences between control and any of the *A. muciniphila*-treated groups (**Figure 1C**). We next assessed the baseline and post-treatment levels of *A. muciniphila*, using species-specific qPCR on fecal DNA. Unexpectedly we found that all controls and treatment groups already carried high levels of *A. muciniphila* at baseline (**Figure 1D**), and oral gavage of *A. muciniphila* failed to modulate the relative abundance of *A. muciniphila* among the treatment groups (**Figure 1D**). Kinetic analysis of *A. muciniphila* abundance found a significant overall decline (*p* < 0.0001) in relative abundance observed between arrival day and all subsequent fecal collection timepoints, with *post-hoc* testing demonstrating significant declines over time across all treatment groups (**Figures 1D, E**). Given the relatively wide variation in *A. muciniphila* abundance among individual mice (**Figure 1D**), we tested for its association with EAE severity. We found no significant association between EAE cumulative disease score and the relative abundance of *A. muciniphila* at any collection timepoint (**Figure 1E**; **Supplementary Figures 1A–E**). Taken together, these results demonstrate that commercially available SPF B6 Jax mice carry high levels of endogenous *A. muciniphila*, which may limit the ability to experimentally manipulate abundance of *A. muciniphila* by oral gavage and hence assess its effect on EAE outcomes in this model.

### Establishment of divergent *A. muciniphila-*free microbiomes allows for stable and reproducible *A. muciniphila* colonization

**Figure 2:**
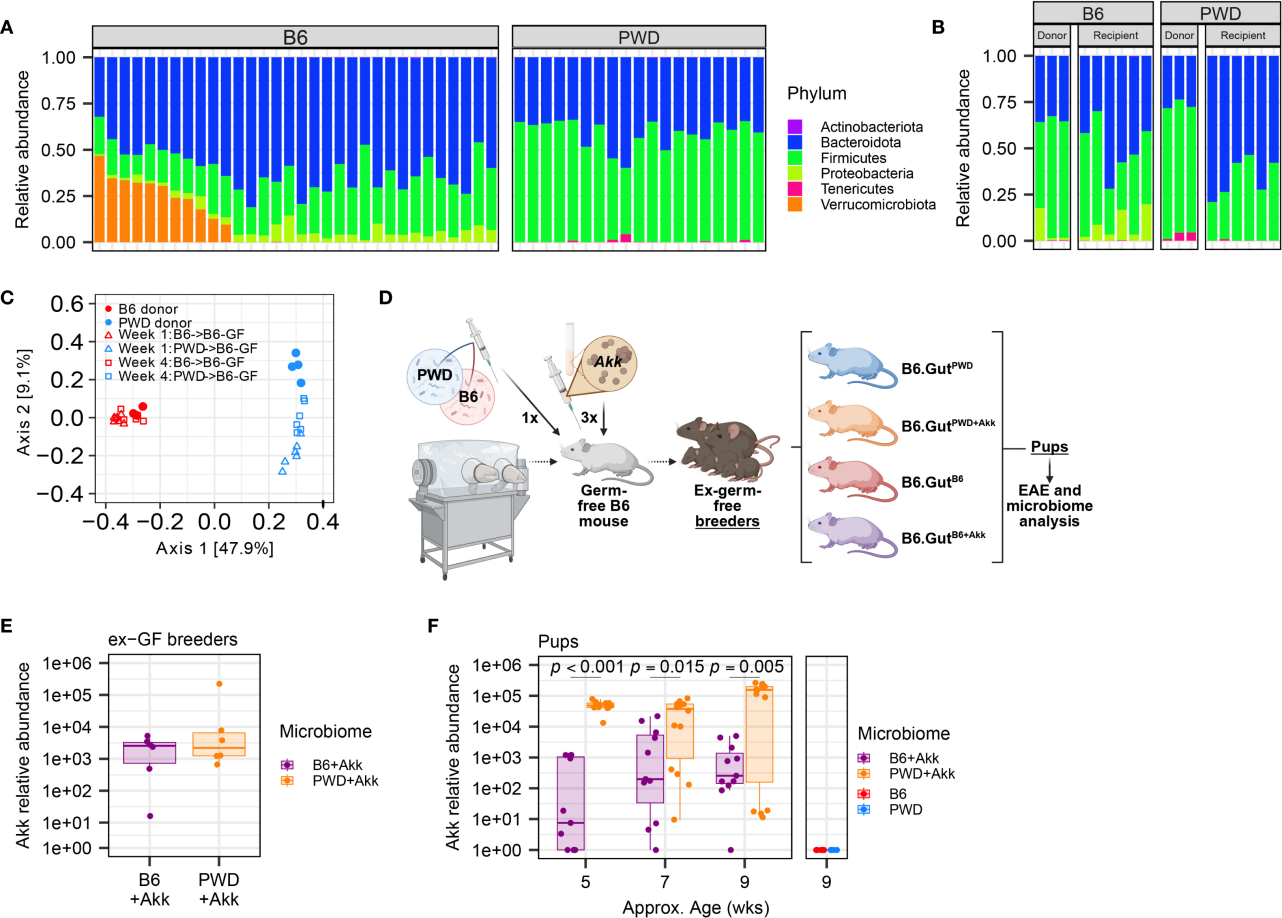
Establishment of two divergent (*A*) *muciniphila*-free microbiomes followed by stable colonization by *A*. *muciniphila*. GF B6 mice were first colonized with cecal stocks from B6 and PWD mouse donors, then colonized by *A*. *muciniphila*, and established as breeding pairs, followed by vertical transmission to offspring. **(A)** Relative abundances by phyla of microbiota from B6 (15 male + 17 female) and PWD (9 male + 10 female) mouse feces as assessed by 16S sequencing; each bar represents an individual mouse. **(B)** Relative abundances of microbiota from B6 and PWD mouse donor ceca and ex-GF B6 recipient feces, 4 weeks after colonization as assessed by 16S sequencing; each bar represents an individual mouse. **(C)** Beta diversity analysis by unweighted UniFrac of gut microbiome composition (assessed by 16S sequencing) of mice shown in **(B, D)** Schematic of experimental design for generating mice used for EAE experiments and microbiome analyses. **(E)** Abundance of *A*. *muciniphila* measured by species-specific qPCR in feces 4 weeks after colonization of 3 pairs of ex-GF B6 breeder mice with B6+Akk and PWD+Akk microbiota (n=6 per microbiome). **(F)** Relative abundance of *A*. *muciniphila* in feces of pups of ex-GF mice from **(E)** and pups of ex-GF B6 mice with (*A. muciniphila*-free) B6 and PWD microbiota (n=5–14 per microbiome-timepoint, includes comparable numbers of male and female mice in each group). Relative abundances from B6+Akk and PWD+Akk microbiota mice were fit to a linear mixed-effects model and assessed by ANOVA, finding a significant effect of microbiome (*p* = 0.00013) and microbiome × age interaction (*p* = 0.0030), with Holm-Bonferroni-adjusted multiple comparisons showing significant differences by microbiome at each timepoint as indicated.

Given that direct administration of *A. muciniphila* to commercially-available mice with an established gut microbiome replete with endogenous *A. muciniphila* failed to appreciably elevate its abundance levels or impact CNS autoimmunity, we pivoted to utilize our previously established compositionally defined microbiome transplantation and vertical transmission model (75, 92). This model capitalizes on cryopreserved cecal microbiota harvested from a set of B6 and genetically divergent Prague wild-derived D (PWD) mice in our own vivarium, allowing the transplantation of these two distinct microbiota contexts into genetically identical germ-free B6 hosts. Using our previously published 16S rRNA DNA sequencing data (75), we first assessed the abundance of endogenous *A. muciniphila* among a large number of B6 and PWD mice in our colony at the time that cryopreserved cecal microbiota stocks were established. We found that *A. muciniphila* (the only representative of the phylum Verrucomicrobiota in these mice) was present in 34% of B6 mice and, surprisingly, completely absent in PWD (**Figure 2A**). We next assessed the abundance of *A. muciniphila* in the cryopreserved B6 and PWD microbiomes, which had been established from a small subset of donor mice from our colony. Serendipitously, both B6 and PWD donor microbiota stocks lacked *A. muciniphila*, which was also recapitulated in the colonized ex-germ-free cecal microbiota transplant recipients (**Figure 2B**), thus providing two divergent *A. muciniphila*-free microbiomes/ecological contexts for our studies.

As previously published (75), we colonized new germ-free B6 recipient mice via oral gavage with stocks of cecal B6 or PWD microbiota, establishing two distinct stably colonized microbiomes, referred to here simply as B6 and PWD, that recapitulated the microbiomes of their respective donor mice and retained the strong divergence in composition between the original donor B6 and PWD microbiomes (**Figure 2C**). Pilot experiments demonstrated that while by 1× gavage or enema of *A. muciniphila* culture was not sufficient to stably colonize *A. muciniphila*-free mice, a 3× gavage regimen was successful (**Supplementary Figures 2A, B**). Thus, we inoculated a subset of B6 and PWD microbiome-colonized ex-germ-free (ex-GF) mice with high-density (1.65×10^8^ CFU) stocks of *A. muciniphila* via oral gavage, successfully colonizing mice following a series of three 200 µl gavages, establishing B6+Akk and PWD+Akk microbiome-colonized ex-GF mice (**Figure 2D**). B6, PWD, B6+Akk, and PWD+Akk microbiome-colonized ex-GF mice were set up as breeding pairs to allow for vertical transmission of their distinct microbiota to their offspring, which were used as experimental animals, thus circumventing potential effects of underdeveloped gut immune-microbe interfaces in ex-germ-free animals (93, 94). Fecal samples from gavage recipient breeders and their offspring were tested for *A. muciniphila* abundance over time, demonstrating stable colonization and vertical transmission of *A. muciniphila* (**Figures 2E, F**). Thus, we were able to generate two distinct microbiome contexts in which we could isolate the effects of stable *A. muciniphila* colonization, while avoiding the confounding effects caused by continuous gavage stress (95). Interestingly, PWD+Akk pups maintained a significantly greater relative abundance of *A. muciniphila* compared to B6+Akk pups, allowing us to leverage microbiome contexts that have both distinct ecological contexts and differing but stable levels of *A. muciniphila* (**Figures 2C, F**).

### Colonization by *A. muciniphila* exacerbates EAE in a microbiome-dependent manner and promotes pro-inflammatory Th17 responses in the CNS

**Figure 3:**
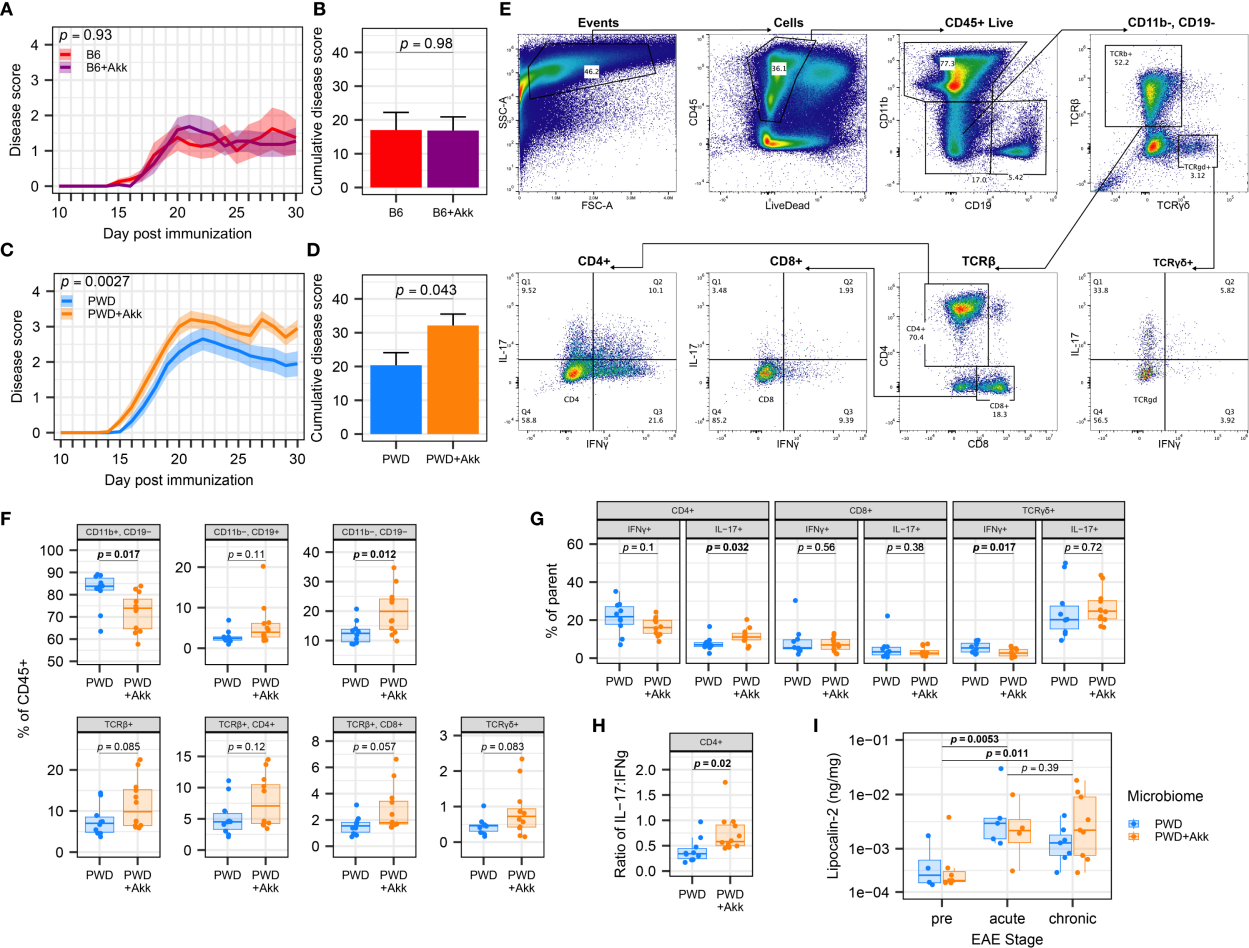
*A. muciniphila*-colonization worsens EAE severity and CNS inflammation in a microbiome-dependent manner. EAE severity was assessed in mice colonized by B6, B6+Akk, PWD, and PWD+Akk microbiomes, with PWD and PWD+Akk representing pooled data from two independent experiments and B6 and B6+ Akk representing one independent experiment. **(A)** EAE course in B6 (n=8, 4 male + 4 female) and B6+Akk (n=11, 6 male+ 5 female) microbiome mice was analyzed by two-way ANOVA, indicating no significant effect *A.* muciniphila colonization (*p* = 0.98) and no significant effect of time × *A*. *muciniphila* interaction (*p* = 0.93). **(B)** Cumulative disease scores of B6 and B6+Akk microbiome mice, analyzed by Student’s t-test. **(C)** EAE course in PWD (n=20, 12 male + 8 female) and PWD+Akk (n=20, 11 male +9 female) microbiome mice was analyzed by two-way ANOVA, indicating a significant effect of *A*. *muciniphila* colonization (*p* = 0.043) and a significant effect of time × *A muciniphila* interaction (*p* = 0.0027). **(D)** Cumulative disease scores of PWD and PWD+Akk microbiome mice, analyzed by Student’s t-test. **(E–H)** Flow cytometric analysis of CNS-infiltrating lymphocytes in PWD (n=10, 5 male + 5 female) and PWD+Akk (n=12, 7 male + 5 female) microbiome mice 30 days after EAE induction (pooled data from two independent experiments). **(E)** Gating scheme for immunophenotyping using concatenated samples. **(F)** Frequencies of the major cell populations as percent of the total CD45^+^ live population, analyzed using Student’s t-tests. **(G)** Intracellular T cell cytokine production assessed as frequencies of parent populations, assessed by Student’s t-tests. **(H)** Ratio of IL-17^+^ to IFNγ^+^ cells among CD4^+^ T cells, assessed by Student’s t-test. **(I)** Fecal lipocalin-2 levels were measured by ELISA, log-transformed, and analyzed by ANOVA of the linear mixed-effects model, finding only a significant effect of EAE stage (*p* = 0.0020) but not microbiome (*p* = 0.93), with Holm-Bonferroni-adjusted pairwise comparisons (n=20 per group, includes males and females), as indicated.

Using our B6, B6+Akk, PWD, and PWD+Akk microbiome-colonized mice, we induced EAE using MOG_35-55_/CFA immunization, as previously described (76). No significant difference was observed between B6+Akk and B6 microbiome mice (**Figures 3A, B**). In contrast, *A. muciniphila* exacerbated EAE severity in PWD+Akk microbiome mice compared to their *A. muciniphila*-free counterparts (**Figures 3C, D**). These data indicate that the effect of *A. muciniphila* on EAE severity is highly dependent on the overall composition of the microbiome into which it is introduced.

To assess whether *A. muciniphila*-mediated EAE exacerbation was associated with augmented CNS-directed immune responses, we isolated immune cells infiltrating into the spinal cord of PWD and PWD+Akk microbiome mice at day 30 post EAE induction for immune phenotyping (**Figure 3E**). Flow cytometry revealed differences in immune profiles in *A. muciniphila-*colonized mice, including a lower frequency of CD11b^+^ myeloid cells and a greater frequency of CD11b^-^CD19^-^ cells, representing additional CNS-infiltrating immune cells, including T cells (**Figure 3F**). *A. muciniphila*-colonized mice also showed a greater frequency of IL-17-producing CD4^+^ T cells, a lower frequency of IFNγ-producing TCRγδ cells, and a trend toward a lower frequency of IFNγ-producing CD4^+^ T cells (**Figure 3G**), which was further substantiated by a significant increase in the ratio of IL-17:IFNγ-producing CD4^+^ T cells (**Figure 3H**). Taken together, these results demonstrate *A. muciniphila* colonization in the PWD microbiome mice leads to a shift towards a pro-inflammatory Th17 phenotype relevant to autoimmune responses and EAE severity.

While PWD mice are not naturally colonized with endogenous *A. muciniphila* (**Figures 2A, B**), our PWD+Akk microbiome mice harbor a high level of *A. muciniphila* (compared to our B6+Akk microbiome mice) (**Figures 2D, E**). Considering that elevated abundance in *A. muciniphila* has been shown in some contexts to induce intestinal inflammation via the degradation of mucin (22, 34), we assessed whether the high abundance of *A. muciniphila* in PWD+Akk microbiome mice was modulating intestinal inflammation prior to and during EAE progression, which by itself has been shown to trigger intestinal inflammation or permeability (96–98). We used fecal lipocalin-2 levels as a sensitive surrogate marker of gut inflammation, as in our previous IBD studies (99) and in EAE studies by others (96). Consistent with previous studies, quantification of lipocalin-2 from fecal samples by ELISA demonstrated significant increases following EAE (**Figure 3I**). However, no significant differences in fecal lipocalin-2 levels between PWD+Akk and PWD microbiome mice were observed either before or following EAE induction (**Figure 3I**). Moreover, no association was found between lipocalin-2 (chronic EAE timepoint) and cumulative disease score (**Supplementary Figure 3A**). Taken together, these results suggest that EAE exacerbation by *A. muciniphila* colonization is not accompanied by significant changes in gut inflammation.

### *A. muciniphila* colonization drives unique changes in the microbiome structure that are highly dependent on baseline microbiome composition, with a depletion in *Clostridia* linked to EAE exacerbation

**Figure 4:**
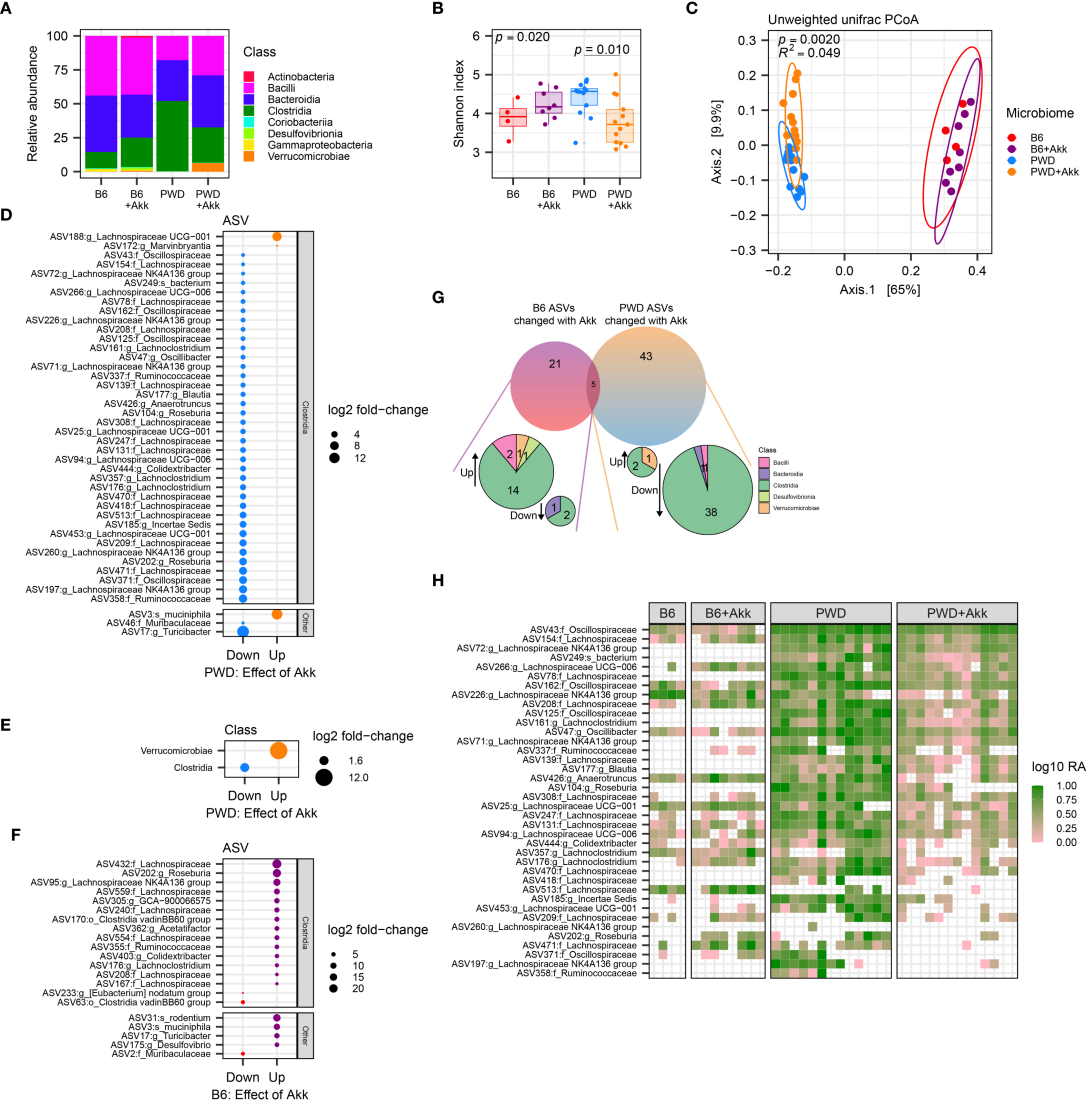
*A. muciniphila* colonization differentially impacts the microbiota in mice colonized with B6 and PWD microbiomes. Pre-EAE microbiota from mice harboring B6 (n=4, 2 males +2 females), B6+Akk (n=8, 4 males +4 females), PWD (n=13, 6 males +7 females)), and PWD+Akk (n=13, 8 males +5 females) microbiomes composition was analyzed by full-length 16S sequencing. **(A)** Relative abundances by class of all bacterial reads in B6, B6+Akk, PWD, and PWD+Akk microbiomes. **(B)** Alpha diversity (Shannon) was analyzed by one-way ANOVA (*p* = 0.020) and multiple comparisons-corrected Wilcoxon tests for B6 vs. B6+Akk (*p* = 0.21) and PWD vs. PWD+Akk (*p* = 0.010) effects of *A*. *muciniphila* colonization. **(C)** Beta diversity (unweighted UniFrac distance) was analyzed by PermANOVA; p- and R^2^ values indicate effect of base microbiome × *A*. *muciniphila* colonization. **(D, E)** Differentially abundant microbiota by ASV **(D)** and bacterial class **(E)** between PWD and PWD+Akk microbiomes. **(F)** Differentially abundant ASVs between B6 and B6+Akk microbiomes. **(G)** Summary of differentially abundant ASVs in B6 and PWD microbiomes driven by *A*. *muciniphila* colonization. **(H)** Heatmap showing log relative abundance of ASVs of the *Clostridia* class that are decreased in PWD microbiome by *A*. *muciniphila* colonization. Each column represents an individual sample.

Our finding that exacerbation of EAE by *A. muciniphila* is highly dependent on the ecological context of the microbiome suggested interactions with other members of the microbiome. We therefore sought to explore how the B6 and PWD microbiomes differ and respond uniquely to colonization by *A. muciniphila*, using full length 16S DNA sequencing to assess gut microbial composition across the 4 microbiomes. We focused our analysis on fecal samples collected prior to EAE induction, in order to avoid potentially confounding effects of EAE progression on gut microbiome composition (35, 57), especially given the differences in EAE severity among groups of interest (**Figures 3A–D**). In agreement with relative abundance determined by qPCR (**Figure 2F**), 16S analysis indicated that *A. muciniphila* represents 6.3% of the total of gut bacterial reads in PWD+Akk microbiome mice compared with 0.64% in B6+Akk microbiome mice (**Figure 4A**; **Supplementary Figure 4A**), confirming successful colonization in both contexts, with relative abundances reflective of those found in human microbiome analyses (23, 100), and indicating a possible difference in niche occupancy across microbiome contexts. Shannon diversity, a metric that considers both species richness and evenness, demonstrated a reduction in alpha diversity driven by *A. muciniphila* colonization only in the PWD microbiome (**Figure 4B**). Statistical analyses of overall microbial community structure (beta diversity) demonstrated a highly significant difference by PermANOVA (*adonis2*) between base microbiomes (B6 vs PWD; *R* ^2^ = 0.64, *p* = 0.001), as expected from our previous studies (75). This analysis also revealed more modest yet significant effects of *A. muciniphila colonization* (*R* ^2^ = 0.052, *p* = 0.004) and of base microbiome × *A. muciniphila* interactions (*R* ^2^ = 0.049, *p* = 0.002), suggesting that *A. muciniphila* colonization alters the composition of the microbiome in a context-dependent manner (**Figure 4C**). Further, using pairwise comparisons on our *A. muciniphila*-free and *A. muciniphila-*colonized microbiomes (83), we found that in the PWD microbiome context, *A. muciniphila* accounted for 6.7% of the Bray-Curtis dissimilarity, but only 0.8% of the dissimilarity in the B6 microbiome context (**Supplementary Figure 4B**). Together, these data suggest that *A. muciniphila* reshapes the PWD gut microbiome to a greater extent than it does the B6 gut microbiome.

In order to identify changes in specific bacterial taxa caused by *A. muciniphila* colonization unique to each of these two different microbiome contexts, we assessed differential abundance of amplicon sequence variants (ASVs) between *A. muciniphila-*colonized mice and their *A. muciniphila-*free counterparts. Analysis of differentially abundant (*p_adj_* < 0.05) ASVs between PWD+Akk and PWD microbiome mice revealed a reduction in the abundance of a large number (40) of ASVs, while only 3 ASVs were increased in abundance, with *A. muciniphila* among them, as expected (**Figure 4D**). Examination of ASVs decreased in abundance by *A. muciniphila* colonization in the context of the PWD microbiome revealed that these ASVs predominantly belonged to the *Clostridia* class, including a number of *Lachnospiraceae, Ruminococcaceae*, and *Oscillospiraceae* family members (**Figure 4D**; **Supplementary Figure 4C**; **Supplementary Table 1b**), all well-known SCFA producers (52). Collapsing the ASVs to the taxonomic level of class confirmed a global reduction in *Clostridia* (**Figure 4E**). These changes suggest that colonization with *A. muciniphila* in the context of the PWD microbiome, where it selectively increases EAE severity, drives a depletion in numerous members of the SCFA-producing *Clostridia* class, prominently including ASVs of the *Lachnospiraceae* family.

We next performed a differential abundance analysis comparing B6+Akk and B6 microbiomes, a context where *A. muciniphila* colonization does not exacerbate EAE. In contrast to changes induced by *A. muciniphila* colonization in the PWD microbiome, this revealed more subtle effects, with fewer (21) total differentially abundant ASVs, with only 3 of these decreased in abundance (**Figure 4F**). The little (5 ASVs) overlap among differentially abundant ASVs between comparisons of B6 ± Akk and PWD ± Akk microbiomes, included the expected increased abundance of *A. muciniphila*, and 4 other ASVs that were decreased in abundance in the PWD ± Akk comparison but increased in the B6 ± Akk comparison (**Figure 4G**). Examination of the differentially abundant ASVs driven by *A. muciniphila* in the B6 context revealed that several *Lachnospiraceae* (11) and/or *Clostridia* (14) ASVs were in fact increased by *A. muciniphila* colonization, while only 2 *Clostridia* ASVs (3 total ASVs) were decreased (**Figures 4F, G**). Taken together, these results demonstrate that colonization by *A. muciniphila* in the context of the B6 microbiome exerts a highly divergent effect compared with colonization in the PWD microbiome, lacking the antagonistic effect on *Clostridia*.

To get at the basis of ASV-specific depletion of *Clostridia* ASVs in the PWD and not the B6 microbiome, we next examined the abundance of the numerous ASVs downregulated by *A. muciniphila* colonization in the PWD microbiome (**Figure 4D**), in the B6 microbiome with or without *A. muciniphila* colonization. We found that many of these ASVs (which were predominantly *Clostridia*) were abundant in the PWD microbiome but either absent (0 mapped reads) or at low abundance in the B6 microbiome with or without *A. muciniphila* (**Figure 4H**), suggesting that in the PWD microbiome, *A. muciniphila* colonization may have unique negative interactions with specific members of the *Clostridia* class, whereas many of these interactions and/or bacteria are absent in the B6 microbiome. Altogether, these findings suggest that *A. muciniphila* colonization exerts highly divergent effects on B6 and PWD microbiome composition, in parallel with divergent effects on EAE severity, suggesting that these two phenotypes are functionally linked.

**Figure 5:**
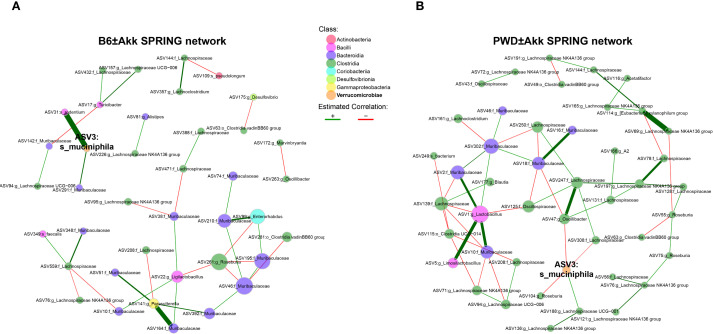
Clostridia networks differ across microbiomes and are uniquely susceptible to disruption by *A*. *muciniphila* colonization. **(A, B)** Semi-parametric rank-based approach for inference in graphical model (SPRING) networks of the B6 ± Akk microbiomes **(A)** and PWD ± Akk microbiomes **(B)**.

Because gut microbiota represent a highly interconnected ecosystem, we utilized an approach to visualize and quantify bacterial interaction networks. We used the semi-parametric ranked-based approach for inference in graphical model (SPRING) approach using the *NetCoMi* package to generate networks of our PWD ± Akk and B6 ± Akk microbiomes, to identify differences in microbial associations and network connectivity. The B6 network had an overall modest connectivity, with a relative largest connected component (LCC) size of 0.44 (**Figure 5A**), compared with the well-connected PWD network, with a relative LCC of 0.86 (**Figure 5B**). Moreover, the B6 network included 18 disconnected subnetwork components compared to 7 components in the PWD network (**Figures 5A, B**), suggesting more cohesion in the microbial community structure of the PWD microbiome. No connectivity between *A. muciniphila* and *Clostridia* was observed in the B6 network (**Figure 5A**). In contrast, the PWD network included numerous negatively weighted edges connecting *A. muciniphila* and various nodes belonging to *Clostridia* (e.g. *Lachnospiraceae*), with the latter also forming many connections with other members of the network (**Figure 5B**). Taken together, these results suggest that the *Clostridia* component of the PWD microbiome represents a well-integrated part of the microbial ecosystem that is particularly vulnerable to disruption by *A. muciniphila* colonization.

### *A. muciniphila* colonization impacts microbial pathways associated with SCFA metabolism and fecal SCFA levels

**Figure 6:**
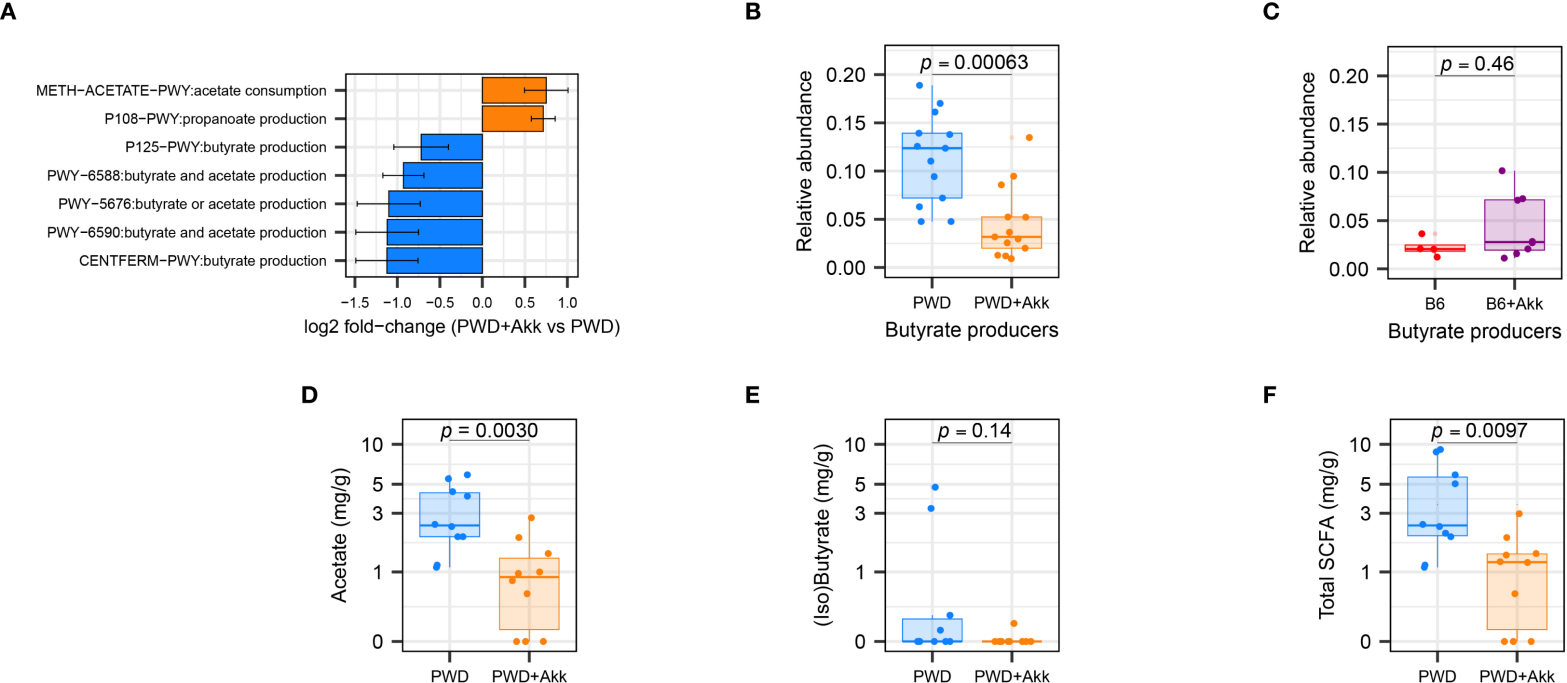
*A. muciniphila* colonization in the PWD microbiome reduces SCFA-producing gut microbiota and their associated metabolites. **(A)** Differentially abundant SCFA-related pathways between PWD and PWD+Akk microbiota, as inferred using PiCRUSt2. **(B, C)** Total relative abundance of ASVs encoding butyrate-producing enzymes in the PWD and PWD+Akk microbiomes **(B)** and B6 and B6+Akk microbiomes **(C)**, with significance of differences assessed by Wilcoxon test. **(D, E)** SCFA quantification from chronic-EAE (D28–32 EAE) timepoint feces of PWD (n=10, 7 males + 3 females) and PWD+Akk (n=10, 5 males + 5 females) microbiome mice showing concentrations of acetate **(D)**, combined isobutyrate and butyrate **(E)**, and total SCFAs **(F)**, analyzed by Student’s t test.

To analyze the functional consequences imparted by *A. muciniphila* colonization across our microbiome models, we employed *PICRUSt2 (* 85) to infer functional microbial gene content from the taxonomic data derived from our full-length 16S analysis. Considering the unique reduction in *Clostridia*-mapped ASVs in the PWD+Akk microbiome, we predicted a shift among pathways pertaining to SCFA production. Indeed, pathway enrichment analysis predicted a reduction in 5 pathways in the PWD+Akk microbiome compared to the PWD microbiome, 2 related to butyrate production and 3 related to both butyrate and acetate production (**Figure 6A**; **Supplementary Table 2b**). Only two SCFA-related pathways, one related to acetate *consumption* and the other related to propionate production, were enriched in the PWD+Akk microbiome (**Figure 6A**). The latter is consistent with the known role for *A. muciniphila* itself in propionate production (22). These results suggest that the selective depletion of *Clostridia* by *A. muciniphila* colonization in the PWD microbiome drives a net depletion in microbial pathways related to SCFA production.

Given these pathway enrichment analysis results, and the reported ability for butyrate to dampen autoimmune responses like those seen in MS (58, 62), we quantified the abundance of ASVs contributing specifically to butyrate production across our microbiome models. As previously published (86), we defined butyrate-producing microbiota as those ASVs whose metagenomes encode terminal butyrate-producing enzymes (87). The relative abundance of total butyrate-producing microbiota was significantly decreased in the PWD+Akk microbiome compared to *A. muciniphila*-free counterparts, whereas butyrate producers showed no such reduction in our B6 ± Akk microbiomes (**Figures 6B, C**). These results confirm the selective depletion of predicted butyrate-producers by *A. muciniphila* colonization in the PWD microbiome.

To determine if the depletion of SCFA-producing microbiota contributed to measurable changes in SCFA levels, we quantified SCFAs in day-30 fecal samples from PWD and PWD+Akk mice via GC-MS. PWD+Akk microbiome mice exhibited a significant depletion of acetate (C2) compared to their *A. muciniphila*-free counterparts (**Figure 6D**), consistent with inferred metagenomic analyses identifying an enrichment in acetate-consuming and a depletion in acetate-producing pathways in the PWD+Akk microbiome (**Figure 6A**). Combined isobutyrate and butyrate levels were also decreased, although not significantly, in PWD+Akk microbiome mice (**Figure 6E**). Total SCFA levels were also significantly decreased in PWD+Akk microbiome mice (**Figure 6F**). Collectively, these data suggest that *A. muciniphila* colonization causes a net decrease in SCFA production in a microbiome composition-dependent manner, which is linked to EAE exacerbation.

### Lack of *A. muciniphila*-mediated EAE exacerbation in the absence of a reduction in *Clostridia* in a different microbiome context

**Figure 7:**
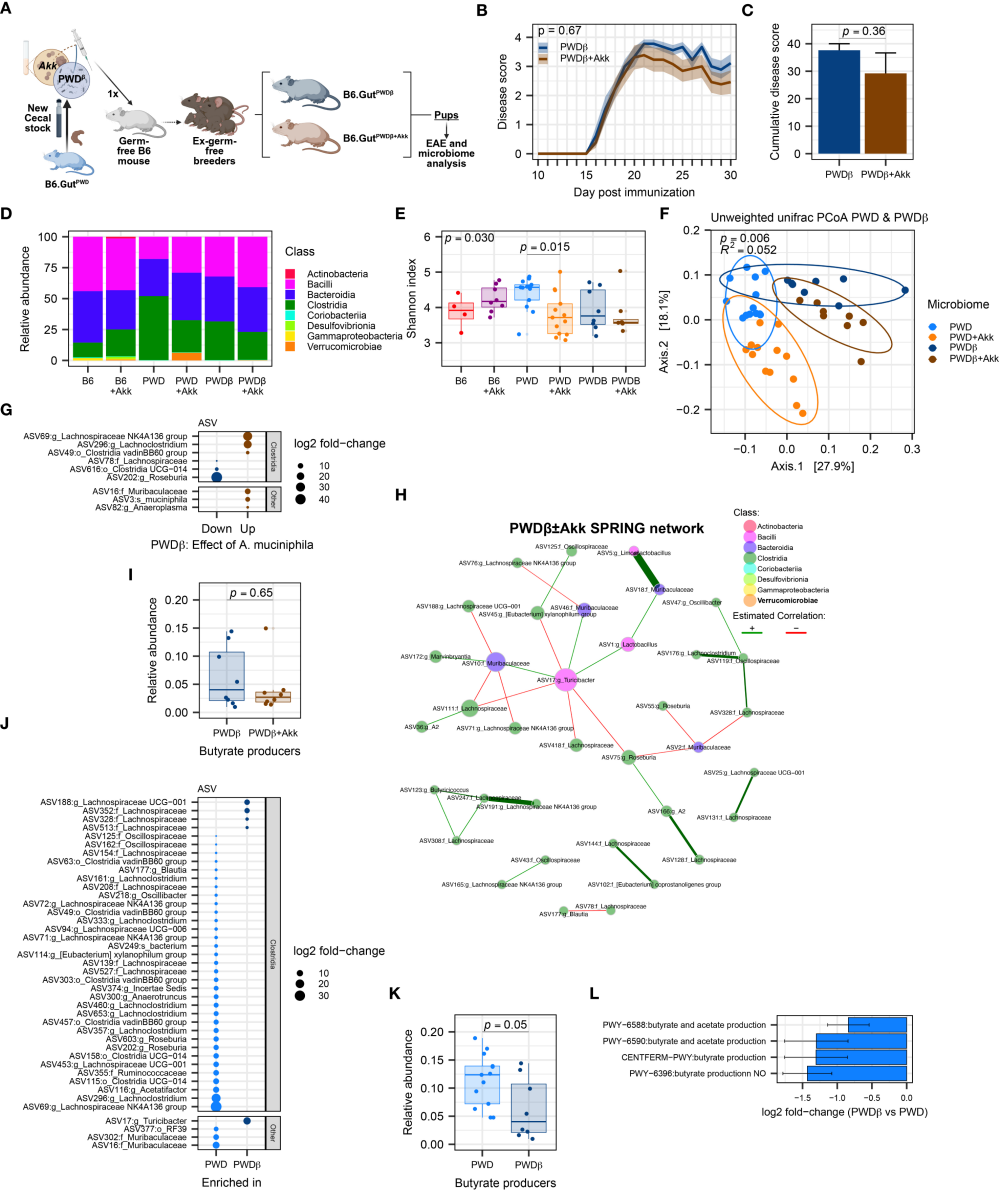
Mice harboring the PWDβ microbiome do not exhibit exacerbated EAE or a reduction in *Clostridia* upon colonization by *A*. *muciniphila*. EAE severity and microbiome composition were assessed in PWDβ and PWDβ+Akk microbiome mice. **(A)** Schematic of experimental design for generating PWDβ microbiome mice used for EAE experiments and microbiome analyses. **(B)** EAE course in PWDβ (n=9, 5 males + 4 females) and PWDβ+Akk (n=13, 7 males + 6 females) microbiome mice, analyzed by two-way ANOVA indicating no significant effect of *A*. *muciniphila* colonization (*p* = 0.36) or time × *A*. *muciniphila* interaction (*p* = 0.67) **(C)** Cumulative disease scores of PWDβ and PWDβ+Akk microbiome mice, analyzed by Student’s t-test (*p* = 0.36). **(D)** Relative abundance of bacterial classes across all 6 microbiomes (PWDβ n=8, 4 males +4 females, PWDβ+Akk n=8, 4 males+4 females), B6 n=4, B6+Akk n=8, PWD n=13, and PWD+Akk n=13). **(E)**. Alpha diversity (Shannon) was analyzed by one-way ANOVA (*p* = 0.030) and multiple comparisons-corrected Wilcoxon test. **(F)** Beta diversity (unweighted UniFrac distance) of PWD, PWD+Akk, PWDβ, and PWDβ+Akk microbiomes was analyzed by PermANOVA; p- and R^2^ values indicate effect of base microbiome × *A*. *muciniphila* colonization. **(G)** Differentially abundant ASVs between PWDβ and PWDβ+Akk microbiomes. **(H)** Semi-parametric rank-based approach for inference in graphical model (SPRING) network of the PWDβ ± Akk microbiomes. **(I)** Total relative abundance of butyrate-producing ASVs in PWDβ and PWDβ+Akk microbiomes analyzed by Wilcoxon test. **(J)** Differentially abundant ASVs between PWD and PWDβ microbiomes. **(K)** Relative abundance of butyrate-producers in the PWD and PWDβ microbiomes, with significance of differences determined by Wilcoxon test. **(L)** Analysis of PiCRUSt2-inferred pathways related to SCFAs production, showing 4 differentially abundant pathways between the PWD and PWDβ microbiomes.

In effort to generate additional B6 mice colonized with the original compositionally well-characterized PWD microbiome using limited original cecal inoculum stocks, we colonized new ex-GF mice using a cecal inoculum from a PWD microbiome-colonized B6 mouse (B6.Gut^PWD^) donor rather than an original PWD donor (**Figure 7A**), generating mice designated as PWDβ microbiome-colonized. To generate *A. muciniphila*-colonized counterparts for these new mice, *A. muciniphila* was combined with the initial cecal inoculum (100µL cecal inoculum + 100µL *A. muciniphila* culture) to generate 3 PWDβ and PWDβ+Akk microbiome breeding pairs whose experimental offspring mice used for EAE (**Supplementary Figure 5A**).

Surprisingly, unlike the effect of *A. muciniphila* seen in the original PWD microbiome-colonized mice, EAE severity did not differ between PWDβ and PWDβ+Akk microbiome mice (**Figures 7B, C**), suggesting that *A. muciniphila* colonization of the original PWD microbiome may have contributed to unique changes in the gut microbiota that were not recapitulated in the context of the new PWDβ+Akk microbiome. To better understand these differences, we analyzed full-length 16S sequencing data from fecal samples of PWDβ and PWDβ+Akk microbiome mice, as above. Surprisingly, *A. muciniphila* represented only 0.20% of the total mapped reads in PWDβ+Akk microbiome mice, more similar to the abundance seen in B6+Akk microbiome mice (**Figure 7D**; **Supplementary Figure 5B**). Comparing the Shannon diversity across all 6 microbiomes demonstrated a significant reduction in microbiome diversity only between PWD and PWD+Akk microbiomes (**Figure 7E**), suggesting that *A. muciniphila* is less disruptive to microbiome diversity in PWDβ compared to the PWD microbiome. To identify differences in the microbial composition among PWD and PWDβ microbiomes, we focused our beta diversity analysis on these microbiomes and their *A. muciniphila*-colonized counterparts (**Figure 7F**). Using PermANOVA, we found a significant effect of base microbiome (PWD vs. PWDβ; *R* ^2^ = 0.23, *p* = 0.001), a significant effect of *A. muciniphila* (*R* ^2^ = 0.14, *p* = 0.001), and a significant *A. muciniphila* × base microbiome interaction (*R* ^2^ = 0.052, *p* = 0.002). These results suggest that the effect of *A. muciniphila* colonization on the overall microbiome composition differs between the PWD and PWDβ microbiomes, with a disparate and muted effect seen in the latter context.

We next performed differential abundance analysis comparing PWDβ and PWDβ+Akk microbiome mice. Compared with the above-described effect in the PWD microbiome (**Figure 4F**), we observed far fewer (9) significantly differentially abundant ASVs (**Figure 7G**) and minimal changes at other taxonomic levels besides *Akkermansia* itself (**Supplementary Figures 5C–E**), confirming a limited effect of *A. muciniphila* colonization on the PWDβ microbiome. In contrast to *A. muciniphila* colonization in the PWD+Akk microbiome (**Figure 4D**), a reduction of the *Clostridia* class was not observed (**Supplementary Figure 5C**), with 2 *Lachnospiraceae* members increased and 2 *Lachnospiraceae* members decreased with *A. muciniphila* colonization (**Figure 7G**). Furthermore, network analysis of the PWDβ ± Akk microbiomes using previously used parameters (**Figures 5A, B**) failed to find any connectivity for *A. muciniphila* (**Figure 7H**), suggesting a lack of major changes to the gut microbiota caused by *A. muciniphila* colonization in the PWDβ microbiome.

To determine the inferred functional effect of *A. muciniphila* in the context of the PWDβ microbiome, we performed pathway enrichment analyses of inferred gene content and quantification of butyrate producer abundance, as above (**Figures 6A–C**). Consistent with the lack of change in overall *Clostridia* abundance between PWDβ and PWDβ+Akk microbiomes, we observed no significant enrichment in pathways related to SCFA metabolism (**Supplementary Table 4C**), with few changes in any functional pathways driven by *A. muciniphila* colonization. Additionally, we observed no significant decrease in the relative abundance of butyrate producers (**Figure 7I**). Taken together, these results demonstrate limited effects of *A. muciniphila* colonization on the composition of the PWDβ microbiome. This suggests the existence of differences between the PWD and PWDβ ecological contexts that limit the ability for *A. muciniphila* colonization to restructure the PWDβ microbiome and contribute to increased EAE severity.

To identify such ecological differences, we compared the base PWD and PWDβ microbiomes by analysis of differentially abundant ASVs, inferred functional pathways, and relative abundance of butyrate producers, as described above. Members of the *Clostridia* class dominated differentially abundant ASVs, with 23 *Clostridia* ASVs underrepresented and 4 *Clostridia* ASVs overrepresented in PWDβ compared to PWD (**Figure 7J**). Moreover, inferred metagenomic analyses identified a significant decrease in in the relative abundance of butyrate producers between these microbiome contexts, emphasizing decreased butyrate production potential in the PWDβ context (**Figure 7K**). Finally, pathway enrichment analysis found 4 SCFA-related pathways, all predicting a reduction in butyrate production potential in the PWDβ microbiome compared with the PWD microbiome (**Figure 7L**). Taken together, these results demonstrate that compared with the PWD microbiome, the derivative but distinct PWDβ microbiome exhibits lower levels of *Clostridia* and SCFA production potential, offering a potential mechanism for the lack of effect of *A. muciniphila* colonization and EAE exacerbation.

### *Clostridia*-rich PWD microbiome promotes suppression of EAE severity by dietary fiber

**Figure 8:**
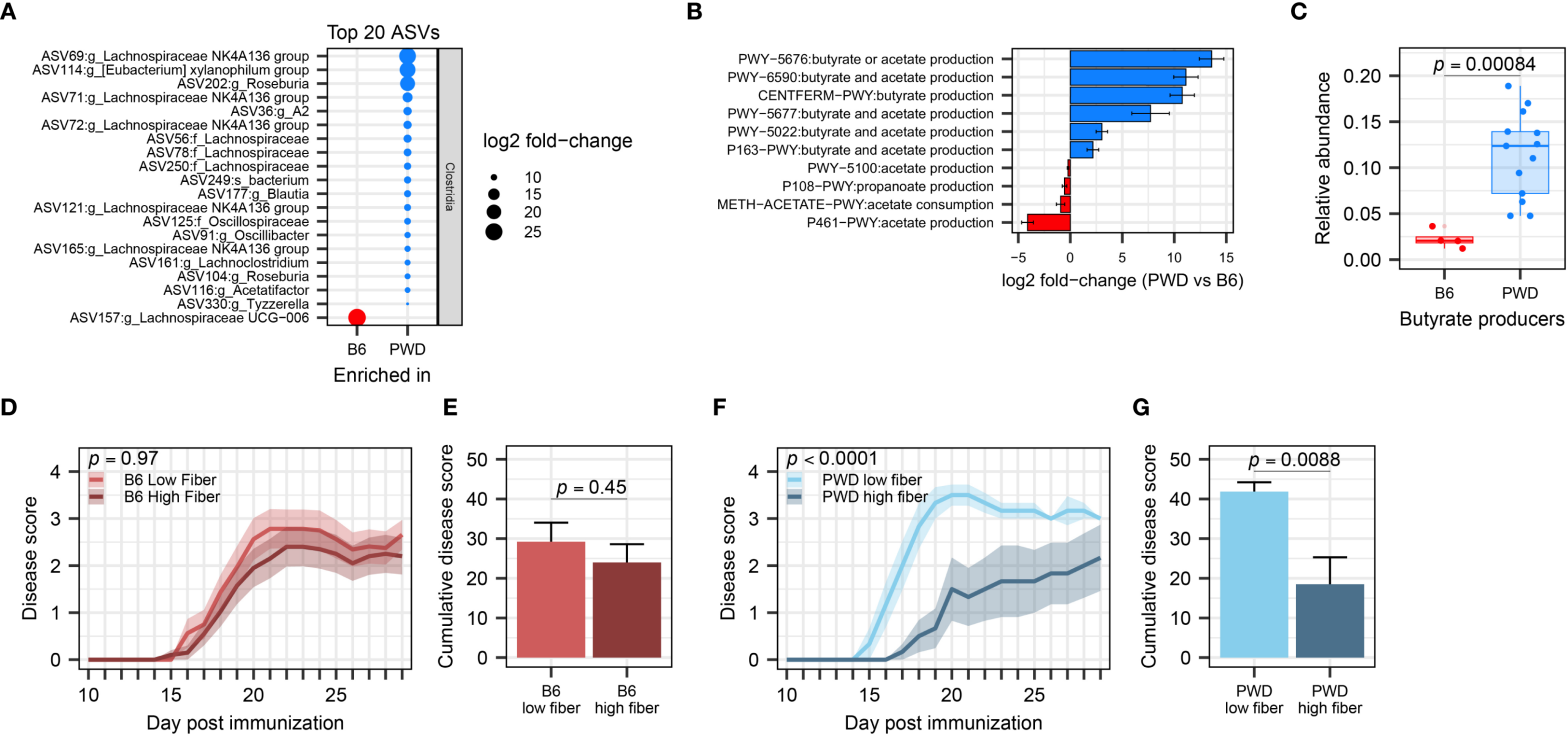
PWD microbiome-colonized mice exhibit enriched abundance of *Clostridia* and efficient suppression of EAE severity by supplementation of dietary fiber. **(A)** Top 20 differentially abundant ASVs of the *Clostridia* class between B6 and PWD microbiomes. **(B)** Pathway analysis related to SCFA production, showing differentially abundant pathways between B6 and PWD microbiota. **(C)** Total relative abundance of butyrate-producing bacteria in the B6 and PWD microbiomes, analyzed by Wilcoxon test. **(D)** EAE course of B6 microbiome mice on low- (n=16, 5 males + 11 females) and high-fiber diets (n=20, 9 males + 11 females), analyzed by two-way ANOVA finding no significant difference by diet (*p* = 0.45) or by time × diet interaction (*p* = 0.97). **(E)** Cumulative disease scores of B6 microbiome mice on low- and high-fiber diets, analyzed by Student’s t-test (p= 0.45). **(F)** EAE course of PWD microbiome mice on low- (n=6, 3 males + 3 females) and high-fiber diets (n=6, 3 males+3 females) analyzed by two-way ANOVA finding significant effects of diet (*p* = 0.0088) and time × diet interaction (*p* < 0.0001). **(G)** Cumulative disease cores of PWD microbiome mice on low- and high-fiber diets, analyzed by Student’s t-test on (*p* = 0.0088).

Because the effect of *A. muciniphila* on EAE exacerbation and *Clostridia* depletion was specific to the PWD microbiome and lacking in the B6 microbiome, we asked whether there was a baseline difference in abundance of *Clostridia* and their associated functional pathways between B6 and PWD microbiomes prior to *A. muciniphila* colonization. Of a total of 136 differentially abundant ASVs, 92 were *Clostridia* members overrepresented in PWD and only 13 *Clostridia* members were underrepresented in PWD (**Figure 8A**; **Supplementary Table 1E**). Notably, when comparing the base PWD and B6 microbiomes for pathways related to SCFA metabolism, 6 pathways related to production of butyrate were significantly overrepresented in the PWD microbiome (**Figure 8B**). Consistent with this, we found butyrate producers to be significantly and dramatically more abundant in the PWD microbiome compared to the B6 microbiome (**Figure 8C**). These results demonstrate that, compared with the B6 microbiome, the baseline PWD microbiome contains more SCFA-producing Clostridia, suggesting a major contribution of SCFA production to the immunological homeostasis conferred by PWD microbiota.

To test the high capacity for, and potential reliance on, SCFA metabolism by gut *Clostridia* in the PWD microbiome in order to maintain immunological homeostasis and prevent autoimmunity, we utilized modulation of dietary fermentable fiber, as a prebiotic substrate for SCFA production. We hypothesized that chow enriched with soluble dietary fiber, which *Clostridia* metabolize to produce SCFAs (101), would attenuate subsequent EAE severity in PWD microbiome mice to a greater extent than in B6 microbiome mice, which have lower abundance of SCFA-producers. PWD and B6 microbiome mice were randomized to high (20% pectin and 10% inulin) and low (0%) fiber diets 2 weeks prior to EAE induction, which were maintained throughout the course of EAE. High fiber diet failed to suppress EAE in B6 microbiome mice (**Figures 8D, E**). In contrast, high fiber diet significantly attenuated EAE severity in PWD microbiome mice (**Figures 8F, G**). Taken together, these results provide functional confirmation that the high SCFA production potential of the PWD microbiome results in a more effective suppression of EAE when excess dietary fiber is provided. They also highlight the importance of baseline microbiome composition in modulating the therapeutic response to prebiotic intervention.

## Discussion

Prior research on the gut microbiome in MS has documented numerous disease-associated microbiota. While informative, these studies have challenges in understanding causative drivers of disease and rely heavily on correlational observations, highlighting the need for longitudinal studies of gut microbiota prior to and after disease onset. In this study, to isolate effects on disease predisposition, we investigated the role of *A. muciniphila* in a model of MS by stably colonizing mice prior to disease onset. Leveraging complex and divergent *A. muciniphila*-free microbiomes, we demonstrated a highly context-dependent exacerbation of EAE by *A. muciniphila* colonization concomitant with a reduction in *Clostridia* and SCFAs. Our study emphasizes the importance of the broader gut microbiome ecological context in modulating functional associations between specific gut microbes and host phenotypes.

Gut microbiota contribute to host immunity through a variety of mechanisms, including changes in barrier homeostasis (102), immunological conditioning (103), and by contributing host-relevant bacterial metabolites (32). Here, we focused on two major signatures of the microbiome found in pwMS: 1) increased abundance of *A. muciniphila* and 2) a reduction in SCFA-producing *Clostridia (* 12–17). In prior literature, probiotic supplementation of *Clostridia* prior to or during the course of EAE has reduced disease. Using a therapeutic approach, human gut-derived *Clostridia* strains were administered via daily gavage starting at the onset of disease disability, leading to lower EAE severity, reduced demyelination, and elevated serum butyrate (58). Similarly, probiotic administration of *Clostridia* three weeks before EAE induction also ameliorated disease severity, reducing lymphocyte infiltration, and demyelination in the spinal cord, as well as reducing Th17 responses and elevating regulatory T cell responses (59). In contrast, both protective and pathogenic effects of *A. muciniphila* on EAE have been reported, with the discrepancy likely dependent on the experimental approach. Two studies from the Weiner group demonstrate alleviation of disease by continuous serial gavage with *A. muciniphila* during disease, with one study initiating treatment prior to disease induction and continuing for 2 weeks after disease induction and the other administering daily gavage treatment for 1 week starting 11 days after disease induction (18, 35). In contrast, two other independent groups have found that stable colonization by *A. muciniphila* worsened EAE severity relative to mice lacking *A. muciniphila*. Mice colonized with a 13-member synthetic human gut microbiota exhibited increased EAE severity (39) and increased fecal lipocalin-2 (33) when also colonized by *A. muciniphila*. In contrast, in the same study, baseline endogenous *A. muciniphila* levels in a complex (SPF) gut microbiome were associated with reduced disease severity (39), echoing our own previous findings across a panel of genetically diverse strains of mice (75). In another (pre-print) study, mice were treated with antibiotics prior to oral administration with cecal contents from *A. muciniphila*-free and *A. muciniphila*-colonized SPF B6 mice, and *A. muciniphila*-colonized mice displayed aggravated EAE severity with elevated IL-17^+^ CD4^+^ T cells in the CNS (104). Antibiotic treatment has also been shown to elevate commensal *A. muciniphila* abundance, altering gut microbiome community structure, and reducing EAE severity (59). When comparing the experimental paradigms between these seemingly contrasting sets of studies, including our own findings, two key factors are likely to account for these divergent results: 1) mode of *A. muciniphila* administration (commensal colonization or probiotic administration) and 2) the ecological structure of the baseline gut microbiome. Studies reporting EAE suppression by *A. muciniphila* broadly used probiotic-like continuous administration throughout disease course. It is notable that in these studies endogenous *A. muciniphila* colonization was not assessed prior to treatment. Consistent with our results in **Figure 1**, commercially available mice can carry high levels of this microbe and existing endogenous baseline colonization of *A. muciniphila* is known to inhibit subsequent experimental engraftment with specific strains of *A. muciniphila (* 105, 106), thus likely influencing effects on the host. In contrast, the studies reporting exacerbation of EAE by *A. muciniphila*, including our own, used stable colonization in an *A. muciniphila*-free baseline, without continuous treatment. We believe that each of these experimental paradigms models a different aspect of host-*A. muciniphila* interactions in MS. The stable colonization approach is more akin to modeling predisposition to MS due to natural colonization by *A. muciniphila* prior to disease onset, which is fully consistent with elevated levels and prevalence of *A. muciniphila* in pwMS compared with healthy controls (12, 13, 15). In contrast, the continuous gavage approach models a probiotic-like therapeutic intervention after disease onset. In this this regard, it is fully consistent with several studies in pwMS, including our own, which have found that elevated *A. muciniphila* levels are paradoxically linked to lower disease severity or progression (18, 21). Moreover, the gut microbiome context-dependent effects of *A. muciniphila* demonstrated here (**Figures 7B, C**), and as supported by divergent outcomes comparing synthetic gut microbiome communities to naturally occurring complex gut microbiomes (107–110), underscore the ecological dynamics as critical in dictating the role of *A. muciniphila* in modulating CNS autoimmunity.

Together, these findings suggest that while endogenous *A. muciniphila* colonization could promote MS predisposition in people at high risk for this disease, paradoxically, probiotic treatment with *A. muciniphila* could be used as an alternative/adjunct DMT in people diagnosed with MS. Related to the latter, our previous longitudinal study in pwMS demonstrated a negative association between *A. muciniphila*-linked vitamin K metabolism and disease progression (21), suggesting that dietary vitamin K intake and levels of vitamin K-producing microbiota should be assessed as important covariates in future experimental and observational studies. Notably, our pathway analysis herein found numerous pathways related to vitamin K production increased with *A. muciniphila* colonization (**Supplementary Table 2B**), which could in fact have been protective in EAE in the context of vitamin K insufficiency. Additionally, *A. muciniphila* can produce several other metabolites that could influence CNS autoimmunity, including an outer membrane protein, Amuc_1100 (111), neurotransmitters (i.e., GABA and serotonin) (112, 113), nicotinamides (114), and polyamines (115, 116), some of which have been shown to be protective against EAE. Moreover, our data highlight the importance of understanding the gut microbial framework within which *A. muciniphila* resides as influencing CNS autoimmune disease, suggesting that microbe-based therapeutic approaches should consider the baseline gut microbiome as a key factor in treatment efficacy. Altogether, our findings suggest that the effect of *A. muciniphila* on MS risk or progression could be modulated by complex inter-microbiota interactions, which are dependent on the microbiome composition that differs across different individuals, cautioning against overinterpretation of changes in abundance of single microbes.

Animal studies suggesting a detrimental effect of *A. muciniphila* have focused on an inflammatory gut mucosal context as a key distinctive factor, ascribing this to thinning of the mucus layer due over-foraging by this mucin-loving microbe (33, 34, 39, 40, 68). In contrast, in our study, we did not find elevated levels of gut inflammatory markers after *A. muciniphila* colonization, suggesting that this is not a major mechanism contributing to EAE exacerbation and echoing the observation that markers of gut permeability and inflammation are not consistently found in pwMS (17, 117, 118). In contrast, an increase in Th17 cells in the CNS by *A. muciniphila* colonization was observed in our own study and Lin et al. (104), suggesting that this could be a major mechanism driving EAE exacerbation. Given the high abundance of Th17 cells in the gut and their ability to traffic from the gut to the CNS in EAE (119, 120), a plausible mechanism would be that *A. muciniphila* promotes generation of gut mucosal Th17 cells, followed by their trafficking to the periphery and priming by myelin antigens. This possibility, as well as the direct and indirect mechanisms by which *A. muciniphila* could induce Th17 cells, should be explored in future studies. It is interesting to note that *A. muciniphila* has been mostly associated with induction of Th1 responses (121), including in PBMCs from pwMS (122). Given these findings, and our findings above (as well as those of others (91, 104)) that the proinflammatory effect of *A. muciniphila* is highly dependent on other members of the microbiota, we find it more likely that *A. muciniphila* drives Th17 responses indirectly, e.g. by depleting SCFA-producing microbes that could drive opposing regulatory responses.

Our study finds a context-specific reduction in predicted SCFA production and butyrate producing bacteria, as well as a decrease in total SCFAs, associated with increased EAE severity driven by *A. muciniphila* colonization. Compared with the somewhat controversial role of *A. muciniphila* in MS, there is a stronger consensus on the beneficial effects of SCFAs and butyrate-producing bacteria in MS (46, 50, 123). Substantial evidence exists for the ability for bacterial-derived SCFAs to regulate T cell differentiation, suppressing autoimmunity and demyelination (45, 62, 124). Specifically, butyrate has been shown to skew T cells away from Th17 responses and towards regulatory phenotypes (44, 125). Although treatment with SCFAs, including butyrate, and butyrate-producing bacteria, has been shown to alleviate EAE in animal studies, SCFA and bacteria supplementation in human populations comes with unique challenges (126, 127). Instead, supplementation with dietary fiber may be a more translatable and tractable approach for pwMS to boost SCFA levels and modulate gut microbiota (34, 86, 128). Our own studies suggest that monitoring of the baseline gut microbiota composition could predict therapeutic responsiveness to such prebiotic interventions, and that *A. muciniphila* abundance could be considered as key co-variate in this context.

### Limitations

The exact mechanisms by which *A. muciniphila* depletes *Clostridia* and drives CNS autoimmunity in a context-dependent manner remain unclear. Previous studies showed bacterial antagonism by *A. muciniphila* has been demonstrated against the Clostridia member *Ruminococcus* via the release of potentially antibacterial metabolites (129). The intestinal mucus layer is a unique niche for gut bacteria, where *A. muciniphila* and *Clostridia* species both reside and interact intimately with each other, potentially in competition for resources like dietary and host polysaccharides and niche occupancy. Such competition could be heightened in microbiome contexts where these mucosal microbiota are more abundant, such as our *Clostridia*-rich PWD microbiome, in which *A. muciniphila* colonizes at a higher abundance (**Figure 2F**). Although we cannot definitively say that the higher abundance of *A. muciniphila* or depletion of *Clostridia* is required for EAE exacerbation, these phenotypes appear to be linked, at least across the three distinct microbiomes examined in our study. This could be addressed in future studies by experimentally increasing the abundance of Clostridia in the B6 microbiome prior to *A. muciniphila* colonization. Moreover, it is formally possible that the high abundance of *A. muciniphila*, rather than the microbial ecology *per se*, drives effects on EAE severity; although the ecology clearly dictates *A. muciniphila* abundance. We also do not know why *A. muciniphila* colonizes the PWD microbiome at a higher level, but this is clearly associated with the baseline abundance of *Clostridia*, and hence potential metabolic crosstalk or even species-specific production of antimicrobial compounds. Nonetheless, research investigating *A. muciniphila* and cancer immunotherapy has suggested a bimodal effect of *A. muciniphila* abundance, where *A. muciniphila*-colonized individuals outperformed *A. muciniphila*-free counterparts, however *A. muciniphila* overabundance (exceeding 4.8%) was associated with reduced overall survival (100). This parameter would describe our PWD+Akk microbiome as having an overabundance (**Figure 6C**; 6.3%) of *A. muciniphila* that may contribute to disease worsening. However, this does not pinpoint how this overabundance contributes to the depletion of *Clostridia*, although it is interesting to note that other classes of gut bacteria are not depleted. To address this, future studies may be able to leverage a reduction in oxygen-sensitive *Clostridia*. In the PWDβ microbiome, this is likely an effect of the secondary cecal microbiota transplant model (**Figure 6A**), contributing to the concurrent loss of EAE responsiveness of this microbiome to *A. muciniphila* colonization. Inter-microbiota interactions remain a complex challenge for elucidating gut microbiota associations with disease, where the use of additional microbiota contexts may become valuable.

While we showed that the effects of *A. muciniphila* on EAE are correlated with a depletion in Clostridia, we did not demonstrate that depletion of Clostridia drives EAE outcomes, e.g. by rescuing EAE exacerbation seen with *A. muciniphila* colonization by supplementation of Clostridia. Moreover, we did not demonstrate that mice colonized with the PWD microbiome and *A. muciniphila* lose responsiveness to high fiber supplementation and SCFA production. These experiments were precluded by the limited quantity of the original PWD microbiome inoculum available to generate new mice for these experiments.

Multiple challenges remain in elucidating the relationship between gut microbiota and MS. The human gut microbiome includes substantial variability across individuals that is poorly captured in animal studies, although our use of divergent gut microbiomes representing two distinct ecological contexts emphasizes the importance in considering microbiota baseline composition in experimental designs. Similarly, changes in bacterial taxonomy and strain differences within bacterial species, especially across host organisms, limits the translatability and reproducibility of gut microbiome research. In this regard, even the full length 16S rRNA sequencing used in our study may underperform in specifying the genera and species of some ASVs compared to shotgun metagenomic methods (130) and only infers gene content and pathways via indirect approaches (131). *A. muciniphila*, previously described as the only species of the *Verrucomicrobiota* phylum to colonize the human gut, now shares its genus with several other *Akkermansia* species, and research suggests that *A. muciniphila* strain variation also contributes differentially towards disease (18). Additionally, the ability for dietary fiber to ameliorate EAE in our *A. muciniphila*-colonized microbiome models remains underexplored, although we would expect blunted amelioration due to the depletion of *Clostridia* seen in PWD+Akk microbiome mice. Because modulating dietary fiber is a highly translatable intervention, interactions between dietary fiber treatment and *A. muciniphila* should be further explored.

## Future directions and conclusions

Given its high abundance and unique metabolic and physical niche in the human gut (132), *A. muciniphila* represents a keystone gut commensal with high potential as a biomarker or therapeutic in autoimmune and/or neurological diseases. However, for this potential to be realized, we must first understand the mechanisms underlying its effects on host physiology. Our study contributes to this body of knowledge by demonstrating that the effects of this microbe on the host are highly dependent on the broader ecological context of the gut microbiome. Specific features of this context, such as high baseline abundance of *Clostridia* or other inter-microbe interactions, that define its susceptibility to microbiome perturbation by *A. muciniphila*, warrant additional functional exploration, e.g. using subtractive approaches with defined minimal microbiota. Unraveling such interactions may be key in addressing disparate effects of *A. muciniphila*’s in MS seen across microbiome studies and mechanisms for harnessing the gut microbiota to dampen disease pathology.

## Data Availability

16S sequencing datasets generated and/or analyzed during the current study are available at the NCBI Sequence Read Archive repository (https://www.ncbi.nlm.nih.gov/bioproject/1336314), accession number: PRJNA1336314.

## Supplementary material

Supplementary Figure 1 A*. muciniphila* abundance does not correlate with EAE severity. SPF B6 Jax mice s received cryopreserved *A. muciniphila* culture, and a control group received vehicle, as indicated in **Figure 2A**. **(A–E)** *A. muciniphila* abundance versus cumulative disease score at various fecal collection timepoints (n=2–8 per group), assessed by linear regression with p-value indicating significance of association and R^2^ indicating goodness of fit.

Supplementary Figure 2 Pilot experiments demonstrate *A. muciniphila* colonization of *A. muciniphila*-free mice following a series of 3 gavages. *A. muciniphila* colonization was assessed in fecal samples by species-specific qPCR. **(A)** Abundance of *A. muciniphila* in PWD microbiome-colonized mice after receiving a single oral gavage of *A. muciniphila* culture or vehicle control (n=7–8 per group, includes comparable numbers of male and female mice). *A. muciniphila* was not detected by species specific qPCR. **(B)** Abundance of *A. muciniphila* in mice after an additional single oral gavage, a single enema, or a series of 3 gavages of cryopreserved *A. muciniphila* culture (n=3–6 per group, includes comparable numbers of male and female mice).

Supplementary Figure 3 Fecal lipocalin-2 levels do not correlate with EAE severity. **(A)** Cumulative disease score and lipocalin-2 concentrations from feces collected during chronic EAE (D29–32 of EAE), analyzed by linear regression finding no significant association.

Supplementary Figure 4 A*. muciniphila* colonization differentially impacts B6 and PWD microbiomes. Microbiota composition was assessed as in **Figure 4**. **(A)** Relative abundance of bacterial reads across all samples; each bar represents an individual mouse. **(B)** Percent contribution to the Bray-Curtis dissimilarity of each individual ASV across B6, B6+Akk, PWD, and PWD+Akk microbiomes, shown as the top 10 ASVs by contribution to each B6 vs B6+Akk and PWD vs PWD+Akk comparison. **(C, D)** Differentially abundant microbiota at taxonomic level of family **(C)** and genus **(D)** between the PWD and PWD+Akk microbiomes. **(E–G)** Differentially abundant microbiota by class **(E)**, family **(F)**, and genus **(G)** between B6 and B6+Akk microbiomes.

Supplementary Figure 5 Colonization by *A. muciniphila* in the PWDβ microbiome leads to minimal compositional changes. **(A)** *A. muciniphila* abundance was quantified by qPCR in the feces of pups of PWDβ+Akk colonized mice (n=7, 5 males + 2 females). **(B–E)** Composition of the indicated microbiomes was assessed by full length 16S sequencing. **(B)** Relative abundance of bacterial reads across all samples and all 6 microbiomes; each bar represents an individual mouse’s gut microbiome composition. **(C-E)** Differentially abundant bacteria at the level of taxonomic class **(C)**, family **(D)**, and genus **(E)** between the PWDβ and PWDβ+Akk microbiomes.

Supplementary Figure 6 Differences between B6 and PWD microbiomes include many taxonomic ranks belonging to the *Clostridia* class. **(A–C)** Differentially abundant bacteria between B6 and PWD microbiomes at the taxonomic rank of class **(A)**, family **(B)**, and genus **(C)**.
