# Supplementary figures and images for: Colonization by Akkermansia muciniphila modulates central nervous system autoimmunity in an ecological context-dependent manner
Source: Front Immunol. 2025 Oct 13;16:1655428. doi: 10.3389/fimmu.2025.1655428 (PMC12557997; doi:10.3389/fimmu.2025.1655428)

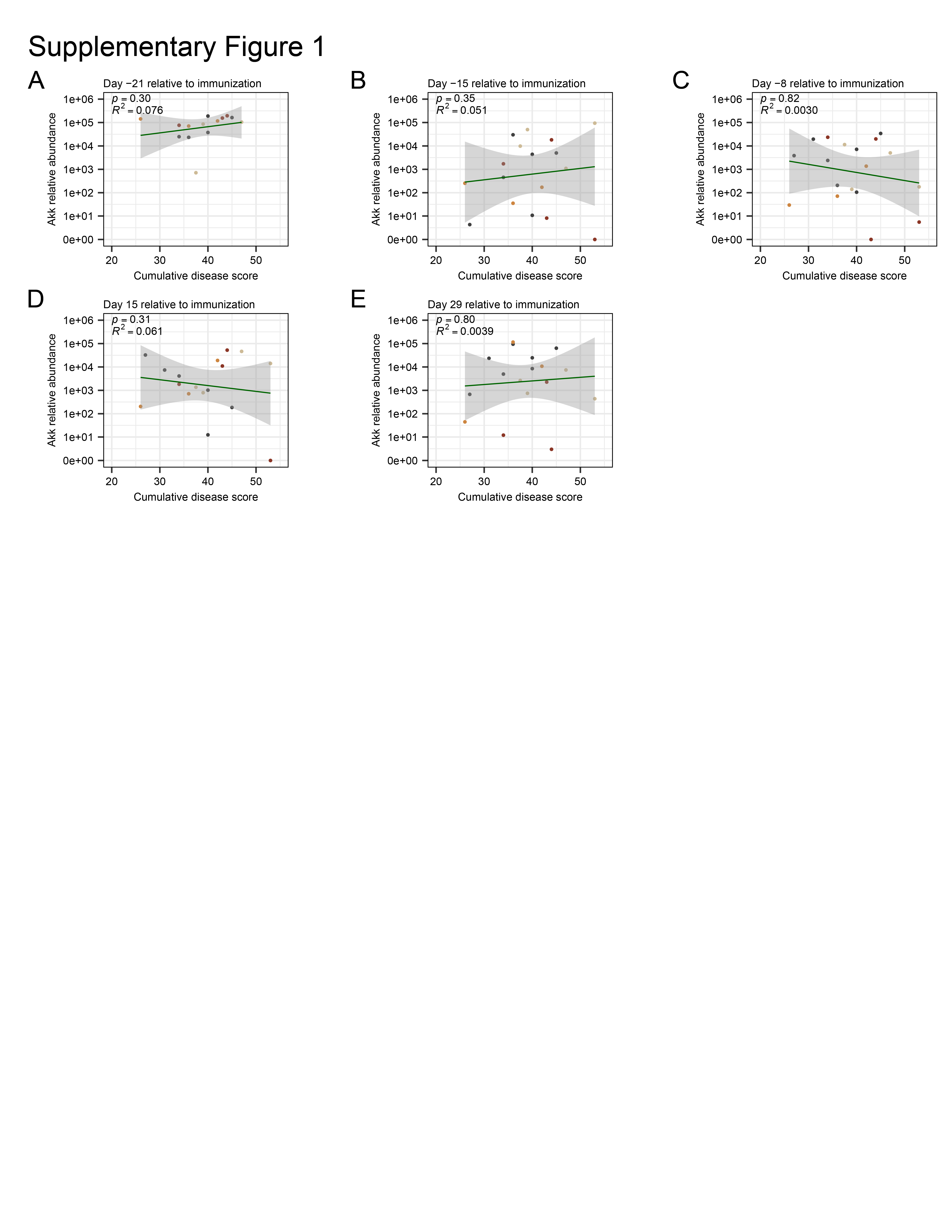

Supplement: Supplementary Figure 1 — A. muciniphila abundance does not correlate with EAE severity. SPF B6 Jax mice s received cryopreserved A. muciniphila culture, and a control group received vehicle, as indicated in Figure 2A . (A–E) A. muciniphila abundance versus cumulative disease score at various fecal collection timepoints (n=2–8 per group), assessed by linear regression with p-value indicating significance of association and R2 indicating goodness of fit. [file DataSheet1.zip › Data Sheet 1 - corrected/Supp. Figure 1.jpg]

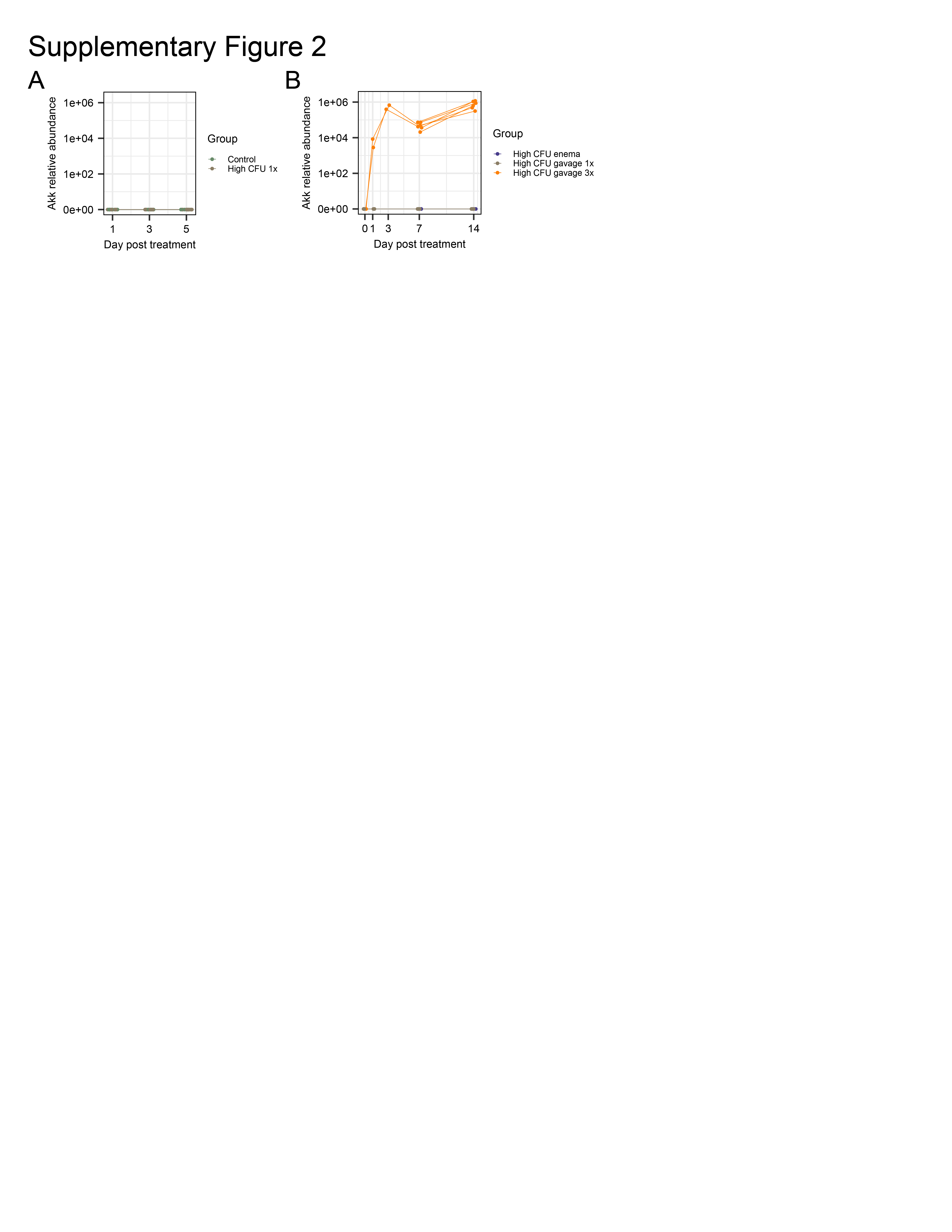

Supplement: Supplementary Figure 1 — A. muciniphila abundance does not correlate with EAE severity. SPF B6 Jax mice s received cryopreserved A. muciniphila culture, and a control group received vehicle, as indicated in Figure 2A . (A–E) A. muciniphila abundance versus cumulative disease score at various fecal collection timepoints (n=2–8 per group), assessed by linear regression with p-value indicating significance of association and R2 indicating goodness of fit. [file DataSheet1.zip › Data Sheet 1 - corrected/Supp. Figure 2.jpg]

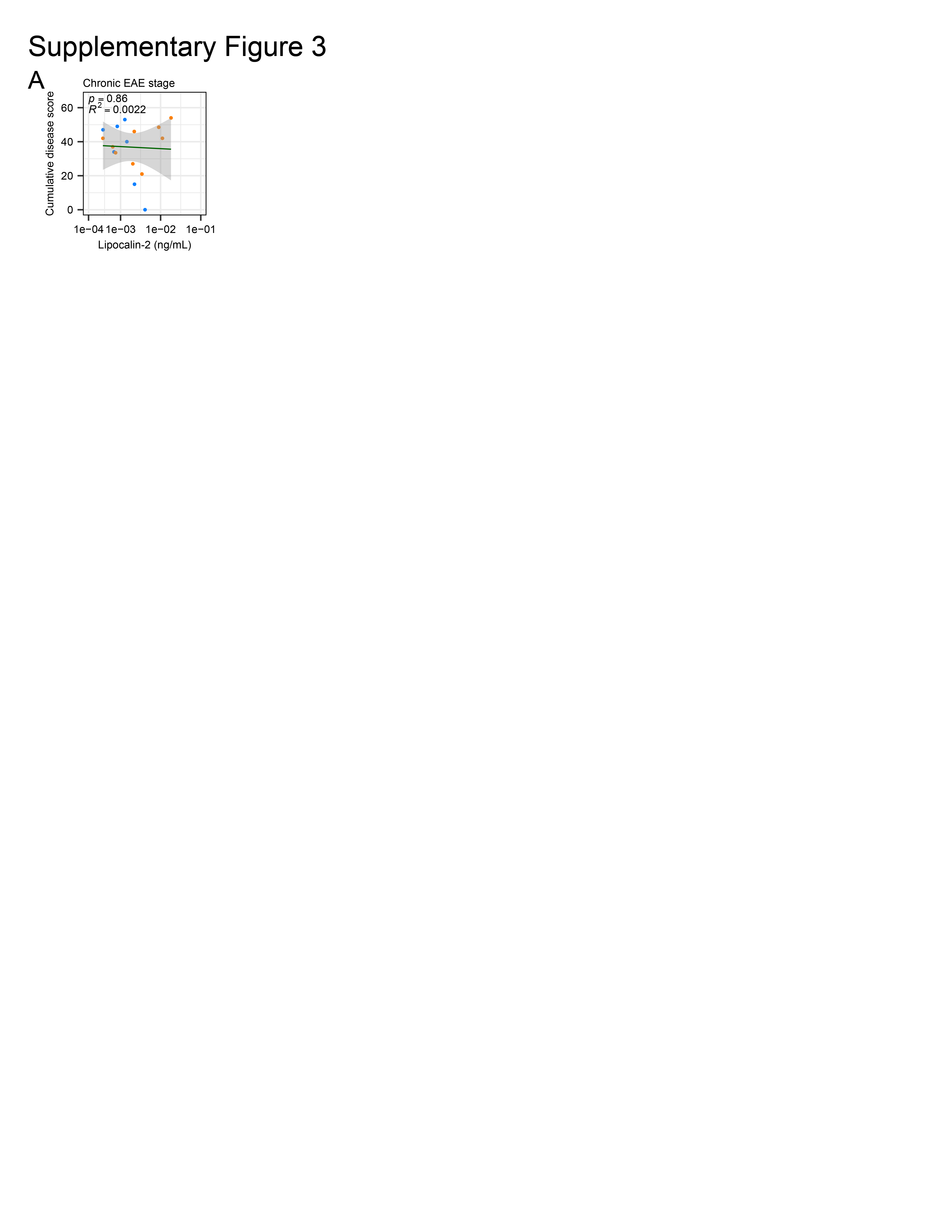

Supplement: Supplementary Figure 1 — A. muciniphila abundance does not correlate with EAE severity. SPF B6 Jax mice s received cryopreserved A. muciniphila culture, and a control group received vehicle, as indicated in Figure 2A . (A–E) A. muciniphila abundance versus cumulative disease score at various fecal collection timepoints (n=2–8 per group), assessed by linear regression with p-value indicating significance of association and R2 indicating goodness of fit. [file DataSheet1.zip › Data Sheet 1 - corrected/Supp. Figure 3.jpg]

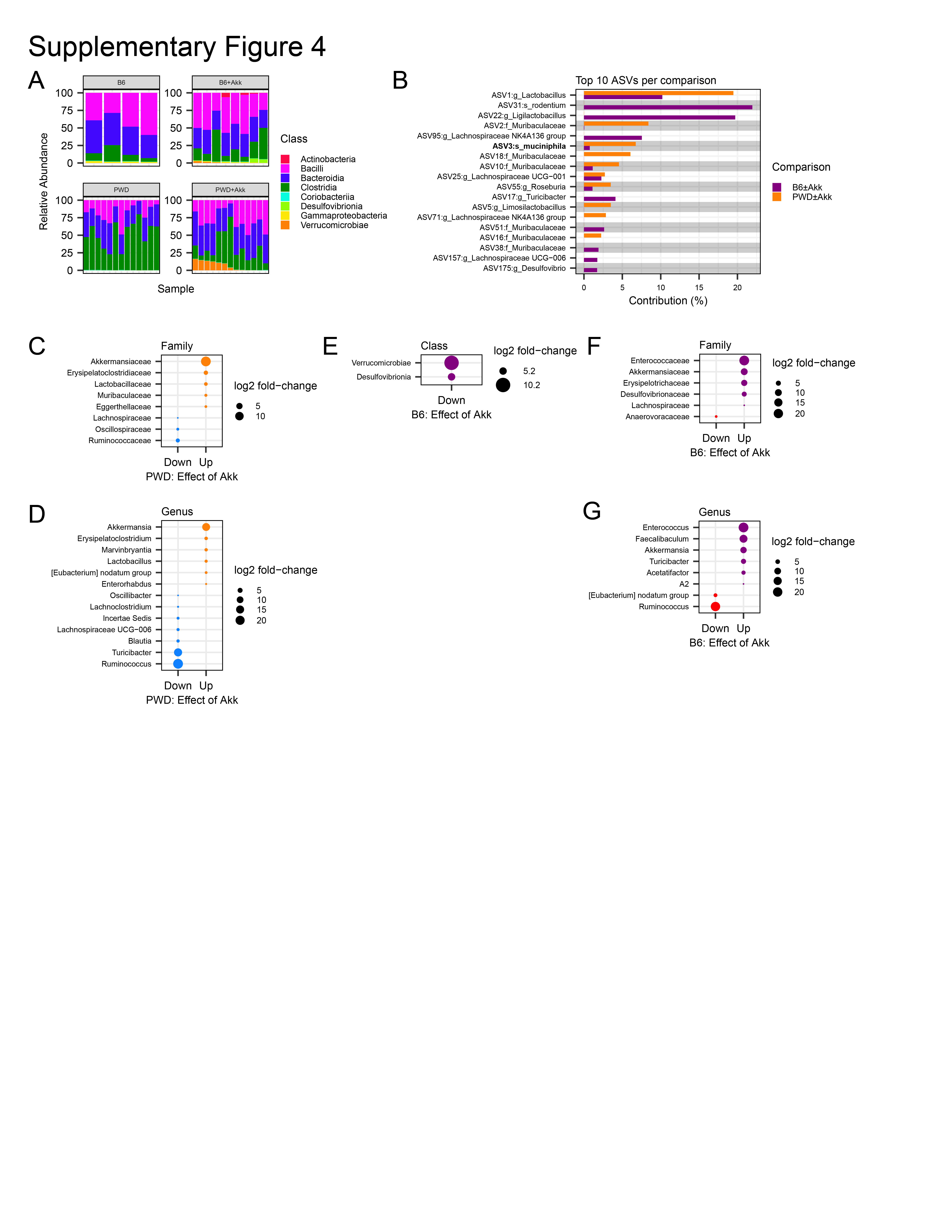

Supplement: Supplementary Figure 1 — A. muciniphila abundance does not correlate with EAE severity. SPF B6 Jax mice s received cryopreserved A. muciniphila culture, and a control group received vehicle, as indicated in Figure 2A . (A–E) A. muciniphila abundance versus cumulative disease score at various fecal collection timepoints (n=2–8 per group), assessed by linear regression with p-value indicating significance of association and R2 indicating goodness of fit. [file DataSheet1.zip › Data Sheet 1 - corrected/Supp. Figure 4.jpg]

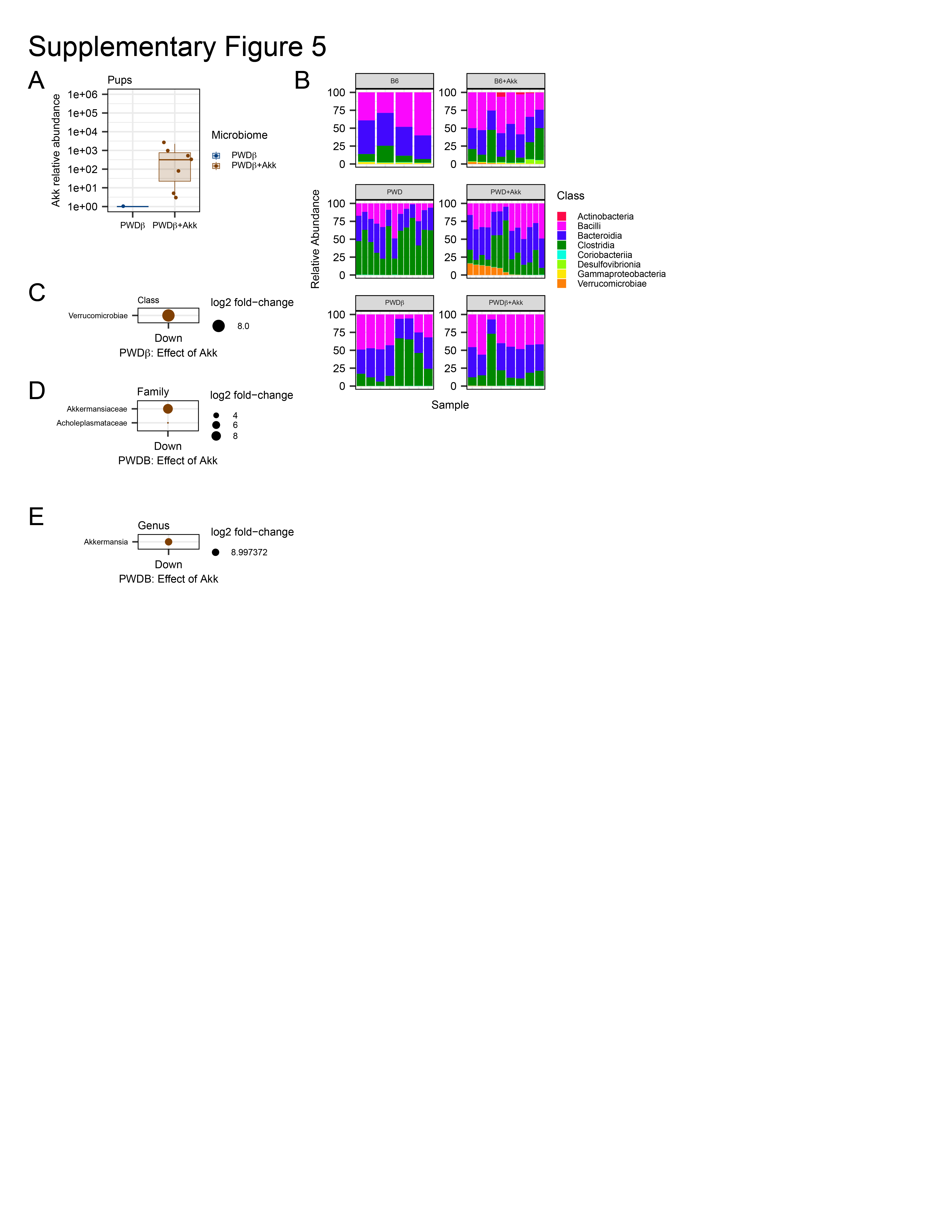

Supplement: Supplementary Figure 1 — A. muciniphila abundance does not correlate with EAE severity. SPF B6 Jax mice s received cryopreserved A. muciniphila culture, and a control group received vehicle, as indicated in Figure 2A . (A–E) A. muciniphila abundance versus cumulative disease score at various fecal collection timepoints (n=2–8 per group), assessed by linear regression with p-value indicating significance of association and R2 indicating goodness of fit. [file DataSheet1.zip › Data Sheet 1 - corrected/Supp. Figure 5.jpg]

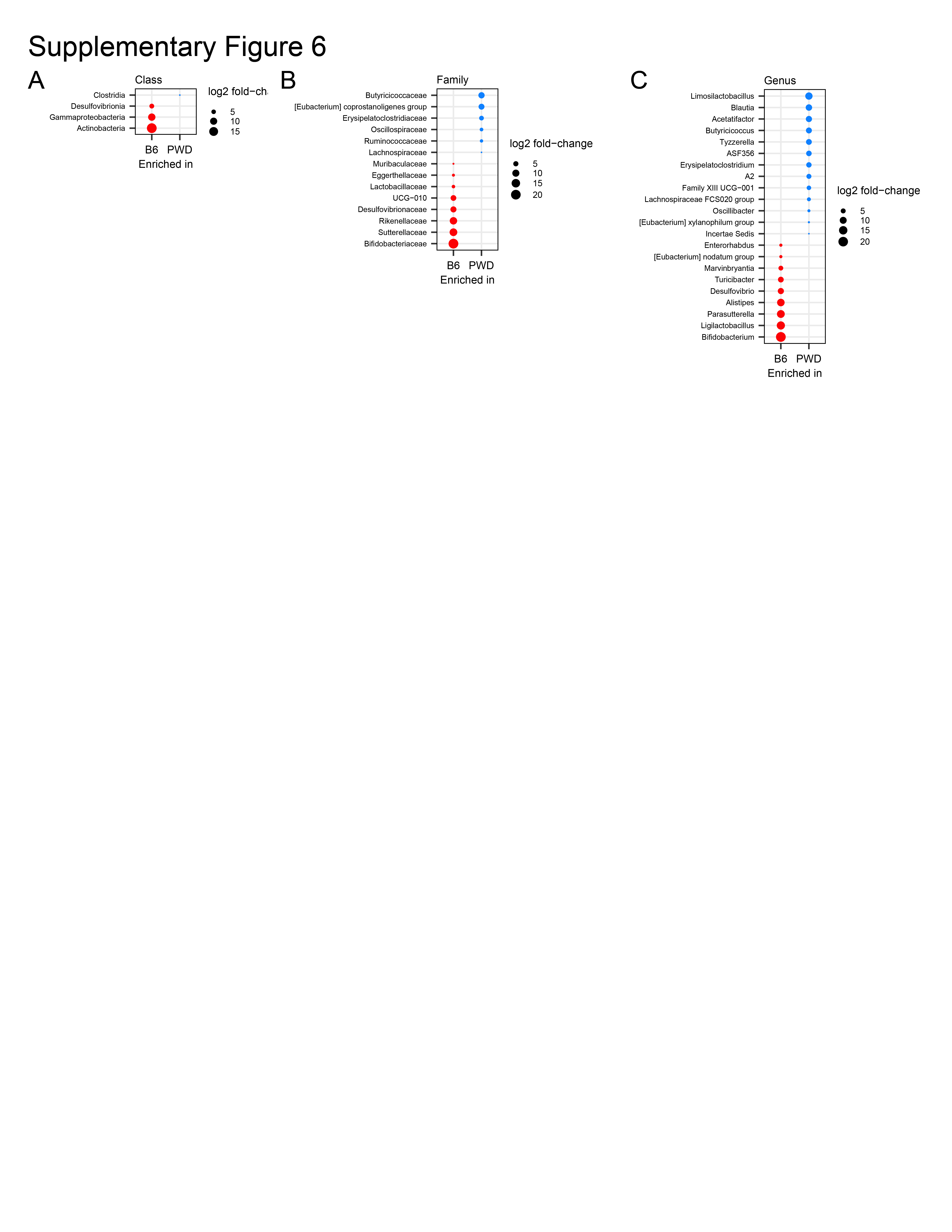

Supplement: Supplementary Figure 1 — A. muciniphila abundance does not correlate with EAE severity. SPF B6 Jax mice s received cryopreserved A. muciniphila culture, and a control group received vehicle, as indicated in Figure 2A . (A–E) A. muciniphila abundance versus cumulative disease score at various fecal collection timepoints (n=2–8 per group), assessed by linear regression with p-value indicating significance of association and R2 indicating goodness of fit. [file DataSheet1.zip › Data Sheet 1 - corrected/Supp. Figure 6.jpg]
